# Modulation of heterologous protein secretion in the thermotolerant methylotrophic yeast *Ogataea thermomethanolica* TBRC 656 by CRISPR-Cas9 system

**DOI:** 10.1371/journal.pone.0258005

**Published:** 2021-09-28

**Authors:** Worarat Kruasuwan, Aekkachai Puseenam, Chitwadee Phithakrotchanakoon, Sutipa Tanapongpipat, Niran Roongsawang

**Affiliations:** 1 Microbial Cell Factory Research Team, Microbial Biotechnology and Biochemicals Research Unit, National Center for Genetic Engineering and Biotechnology, National Science and Technology Development Agency, Khlong Nueng, Khlong Luang, Pathum Thani, Thailand; 2 Microbial Systems and Computational Biology Research Team, Thailand Bioresource Research Center, National Center for Genetic Engineering and Biotechnology, National Science and Technology Development Agency, Khlong Nueng, Khlong Luang, Pathum Thani, Thailand; CNR, ITALY

## Abstract

The thermotolerant methylotrophic yeast *Ogataea thermomethanolica* TBRC 656 is a potential host strain for industrial protein production. Heterologous proteins are often retained intracellularly in yeast resulting in endoplasmic reticulum (ER) stress and poor secretion, and despite efforts to engineer protein secretory pathways, heterologous protein production is often lower than expected. We hypothesized that activation of genes involved in the secretory pathway could mitigate ER stress. In this study, we created mutants defective in protein secretory-related functions using clustered regularly interspaced short palindromic repeats (CRISPR)–CRISPR-associated protein 9 (Cas9) tools. Secretion of the model protein xylanase was significantly decreased in loss of function mutants for oxidative stress (*sod1*Δ) and vacuolar and protein sorting (*vps1*Δ and *ypt7*Δ) genes. However, xylanase secretion was unaffected in an autophagy related *atg12*Δ mutant. Then, we developed a system for sequence-specific activation of target gene expression (CRISPRa) in *O*. *thermomethanolica* and used it to activate *SOD1*, *VPS1* and *YPT7* genes. Production of both non-glycosylated xylanase and glycosylated phytase was enhanced in the gene activated mutants, demonstrating that CRISPR-Cas9 systems can be used as tools for understanding *O*. *thermomethanolica* genes involved in protein secretion, which could be applied for increasing heterologous protein secretion in this yeast.

## Introduction

Yeast is widely used as a cell factory for the production of heterologous proteins. The thermotolerant methylotrophic yeast, *Ogataea thermomethanolica* TBRC 656 [1,2], is a non-conventional yeast which has been demonstrated as an alternative cell factory for the production of heterologous proteins [3]. This yeast can utilize methanol or sucrose to drive the inducible expression of recombinant proteins [4,5]. In addition, it can be cultured to high-cell density on fed-batch fermentation using low-cost substrates, which is advantageous for industrial applications [6,7]. Like other yeasts, efficient secretion is a major bottleneck for heterologous protein production, in which heterologous proteins are often poorly secreted, leading to intracellular accumulation, which triggers endoplasmic reticulum (ER) stress [8,9]. Therefore, improving the secretory capacity through the engineering of secretory pathways could improve protein production, for example, by the fusion of signal peptides to the heterologous protein, by the engineering of protein folding, and by the ER quality control and engineering of vacuolar sorting pathways [10,11].

Yeast protein secretion comprises several complex steps mediated by numerous cellular proteins to transport proteins to the external medium, which can be divided into protein translocation across the ER membrane, protein folding and maturation in the ER and vesicular transport or protein trafficking. In general, eukaryotic protein trafficking from the Golgi can go via several routes after the Golgi maturation processes are completed. Proteins are either recycled back to the Golgi, traversed through the plasma membrane for exocytosis, or transferred to the vacuole for further storage or degradation [12]. The carboxypeptidase Y (CPY) pathway is an indirect transport pathway to vacuoles via the early endosome or multivesicular body [13]. In order to deliver the cargo to the vacuole, proper fusion of the interacting membranes has to occur. Ras-associated binding GTPases (Rab) function in multiple steps of membrane trafficking, including protein sorting and vesicle transport [14]. For the fusion process of yeast vacuoles, the homotypic fusion and vacuole protein sorting (HOPS) complex is initially associated with soluble N-ethylmaleimide-sensitive factor attachment protein receptors (SNAREs) and then complexed with Rab GTPase Ypt7 for tethering and vacuole membrane fusion [15,16].

Apart from these complexes, the dynamin-like Vps1 protein is also reported to be involved in vacuolar membrane homeostasis. Vacuolar protein sorting-associated protein 1 (*VPS1*) is a pre-vacuolar secretory gene that encodes a GTPase which mediates the budding of clathrin-coated vesicles from the late Golgi [17]. Furthermore, Vps1 and Ypt7 cooperate with retromer in a major protein complex for recycling of membrane proteins to the Golgi and plasma membrane in order to maintain the functionality of the vacuole [18]. Yeast cells lacking *vps1* are reported to be not defective in endocytosis but instead secrete the Golgi-modified form of CPY and transport vacuolar membrane proteins to the plasma membrane [19]. Additionally, *vps1*-disrupted *Schizosaccharomyces pombe* is also reported to be less tolerant of oxidative stress triggered by high concentrations of Ca^2+^ and Fe^2+^ [20]. Superoxide dismutase (Sod) is a reactive oxygen species (ROS) scavenger enzyme that modulates ROS in the cell by catalyzing the dismutation of superoxide anions (O_2_^−^) into oxygen and hydrogen peroxide [20,21]. *Kluyveromyces lactis SOD1* gene overexpression reduces ROS, which increases cell proliferation and protein production [21]. Hence, modulation of ROS caused by ER-stress is an alternative way to improve the protein production of heterologous protein and increase the secretion capacity.

In recent years, clustered regularly interspaced short palindromic repeats (CRISPR)–CRISPR-associated protein (Cas) systems have been employed as RNA-guided endonuclease technologies for genome engineering in many organisms [22], with CRISPR-Cas9 the most widely used [23]. CRISPR-Cas9 genetic tools have been developed for mutagenesis in non-conventional yeasts including *K*. *lactis*, *K*. *marxianus*, *Ogataea polymorpha*, *O*. *thermomethanolica* and *Yarrowia lipolytica* [24–26]. Instead of mutagenesis, it is possible to create mutants by modulating gene expression with endonuclease-inactive Cas9 mutant (dCas9) [27]. By tethering dCas9 to a transcription activator, sequence-specific activation of target gene expression (CRISPRa) can be accomplished in yeast cells [28,29].

Efficient secretion is crucial for heterologous protein expression in yeast hosts. However, the protein secretory capacity of yeast is often insufficient, leading to lower than expected protein secretion and also ER stress by accumulation of unfolded proteins in the ER [8]. Yeast mitigate ER stress via the unfolded protein response (UPR), which is induced by the transcriptional activator Hac1 [30]. The *O*. *thermomethanolica HAC1* gene homolog was disrupted and the resultant null mutant exhibited lower expression of secretory pathway proteins that function in glycosylation, protein transport, vacuole and protein sorting. Examples included Atg12 (-2.7 fold), Atg18 (-4.7 fold) and Vps1 (-9.7 fold). However, expression of the oxidative stress protein (Sod1) was upregulated 44.4-fold in the mutant [31]. In addition, a comparative proteomic approach identified several proteins with altered expression during heterologous protein secretion in *O*. *thermomethanolica*. Mutations of genes differently expressed during heterologous protein secretion, including genes involved in vacuole and protein sorting (*COF1*), cell wall biosynthesis (*GAS4*), chaperone (*PEX19*) and protein transport (*YPT35*) showed altered xylanase secretion compared with the control strain [32]. Although these studies highlight genes potentially involved with mitigating the negative effect of heterologous protein secretion, there is little information available about pathways that are crucially responsible for protein secretion in *O*. *thermomethanolica*. Therefore, oxidative stress and vacuole and protein sorting related genes were chosen as genome engineering targets. In this study, we created *O*. *thermomethanolica* mutants with the aim of improving protein secretion capability by using non-glycosylated fungal xylanase and glycosylated fungal phytase as model heterologous proteins, which are routinely used in the feed and fuel industries [33]. Five genes involved in the secretory pathway, *ATG12*, *ATG18*, *SOD1*, *VPS1* and *YPT7*, were mutated by CRISPR-Cas9 and CRISPRa was tested for activation of protein secretion genes.

## Materials and methods

### Strains and culture conditions

All strains used in this study are described in Table 1. Strain Ot-Mal-Xyl and Ot-Mal-Phy, previously constructed by transformation of either pOtMal-Xyl or pOtMal-Phy plasmids into *O*. *thermomethanolica* TBRC 656 wild-type [5], were used as a host for yeast transformation. All engineered yeast strains were cultivated on YPD medium (10 g L^–1^ yeast extract, 20 g L^–1^ peptone, and 20 g L^–1^ glucose) supplemented with appropriate antibiotics at 30°C with 250 rpm shaking. *Escherichia coli* strain DH5α was used to propagate plasmids and was cultured in Luria-Bertani (LB) broth with appropriate antibiotics at 37°C with 200 rpm shaking.

**Table 1:** Strains and plasmids used in this study.

Strains or plasmids	Relevant characteristics	Source
**Strains**		
*E*. *coli* DH5α	Commercial host for cloning	
Ot	*Ogataea thermomethanolica* wild-type strain TBRC 656	[1,2]
Ot-Mal-Xyl	Ot harboring fungal xylanase gene from *Aspergillus niger* BCC14405 under the control of pMal promoter, Zeo^R^	[5]
Ot-Mal-Phy	Ot harboring fungal phytase gene from *Aspergillus niger* BCC18313 under the control of pMal promoter, Zeo^R^	[5]
Ot-Cas9-Xyl	Ot harboring pOtAOX-Cas9 and fungal xylanase gene under the control of pMal promoter	[25]
Ot-dCas9-VP64-Xyl	Ot harboring pOtAOX-dCas9-VP64 and fungal xylanase gene under the control of pMal promoter	This study
Ot-dCas9-VP64-Phy	Ot harboring pOtAOX-dCas9-VP64 and phytase gene under the control of pMal promoter	This study
**Plasmids**		
pOtMal-Xyl	pOtMal expressing xylanase, Zeo^R^	[5]
pOtMal-Phy	pOtMal expressing phytase, Zeo^R^	[5]
pTPGI_dCas9_VP64	pTPGI expression vector containing dCas9 gene with N-terminally tagged with SV40 nuclear localization signal and C-terminal fused with VP64, TRP1^R^	[29]
pPasNeo-OTOT	pOtAOX inducible expression vector with Neo^R^	[25]
pOtAOX-Hyg	pOtAOX inducible expression vector with Hyg^R^	[25]
pOtAOX-Cas9	Inducible expression vector containing Cas9 gene under the control of pOtAOX promoter and Neo^R^	[25]
pOtAOX-dCas9-VP64	Inducible expression vector containing dCas9 gene under control of pOtAOX promoter with N-terminally tagged with SV40 nuclear localization signal and C-terminal fused with VP64, Neo^R^	This study
pOtAOX-gRNA	Inducible expression vector containing gRNA cassettes (HH–20 bp specific determinant sequence–structural gRNA–HDV) under control of pOtAOX promoter and Hyg^R^	This study

### Defining targets of the UPR

To test whether the expression of five genes involved in the secretory pathway changes in response to ER stress as path of the UPR, wild-type *O*. *thermomethanolica* was cultivated to an initial OD_600_ = 1.0 in YPD at 30°C with 250 rpm shaking for 8 h. After that, the mid-log phase cell cultures were then treated with 5 mM dithiothreitol (DTT), a known ER stressor, for 60 min. Transcriptional targets of the UPR were analyzed by quantitative real-time PCR analysis using specific primers for each target gene as described in Table 2.

**Table 2:** List of primer sequences used in this study.

Description	Name	Sequence (5’-3’)	Source
**dCas9-VP64 amplification**			
	dCas9-F	ATATAAGCTTATGTCTAGAGCCCCAAAGAAG	This study
	dCas9-R	ATATGAATTCCTAGGAAAGCATGTCTAAGTC	
**Site-specific of pOtAOX-dCas9-VP64**			
	Int-pAOX-F	ATTGCACGACGTTCATCCTC	This study
	Int-dCas9-VP64-R	ACTTCTTGGATGGAACCTTG	
**pOtAOX-gRNA integration**			
	OtAOX-F	CCAATGCATGCACAAGCTGGACGAGTCGC	This study
	OtAOX-R	GCAAATGGCATTCTGACATCC	
**Hygromycin resistance gene amplification**			
*Hyg* ^R^	Hyg-F	ATGAAAAAGCCTGAACTCACC	This study
	Hyg-R	TCCATCACAGTTTGCCAGTG	
**Single gene mutagenesis**			
*ATG12*	Atg12-F	CCTCAATCAATAGCGGAAGG	This study
	Atg12-R	ACAATAAGCTCTTCACCCACA	
*ATG18*	Atg18-F	CAAATCCACTTGCCTTCGAG	This study
	Atg18-R	GAGGGAGAAAGCGATACCAA	
*SOD1*	Sod1-F	GGAGACTCCACTGTCAAGGG	This study
	Sod1-R	TCAAGCAGGAAAGCCAATG	
*VPS1*	Vps1-F	ATGGATGAAACATTAATCCAAACC	This study
	Vps1-R	TTGGTCAGTCCAGGCAAATC	
*YPT7*	Ypt7-F	GCAAATATGGGACACTGCTG	This study
	Ypt7-R	CTGAACTCGAGACTCTCCTC	
**RT-qPCR analysis**			
*ACT*	RT-Act-F	CTTTCAACGTTCCAGCTTTC	This study
	RT-Act-R	AGGAACAACGTGGGTAACAC	
*ATG12*	RT-Atg12-F	CTTAGCGAGCTCCTTGATGG	This study
	RT-Atg12-R	ATCATGCTCTGGTTGAGGGT	
*ATG18*	RT-Atg18-F	TGCAAGCGACAAAGGAACAA	This study
	RT-Atg18-R	ATTTGCGGCTTCTTCCTCAC	
*SOD1*	RT-Sod1-F	CCACTGTCAAGGGAATTGTT	This study
	RT-Sod1-R	AATTGATGGATGTGGAAACC	
*VPS1*	RT-Vps1-F	CCAATGAGTTGAGTGGAGGT	This study
	RT-Vps1-R	GTCTGCATCCTTGATCTGGT	
*YPT7*	RT-Ypt7-F	TTGTGTACGATGTCACCAAC	This study
	RT-Ypt7-R	CAAACGGGAAGTTATCAGGA	

### Construction of plasmids and engineered yeasts

**Fig 1:**
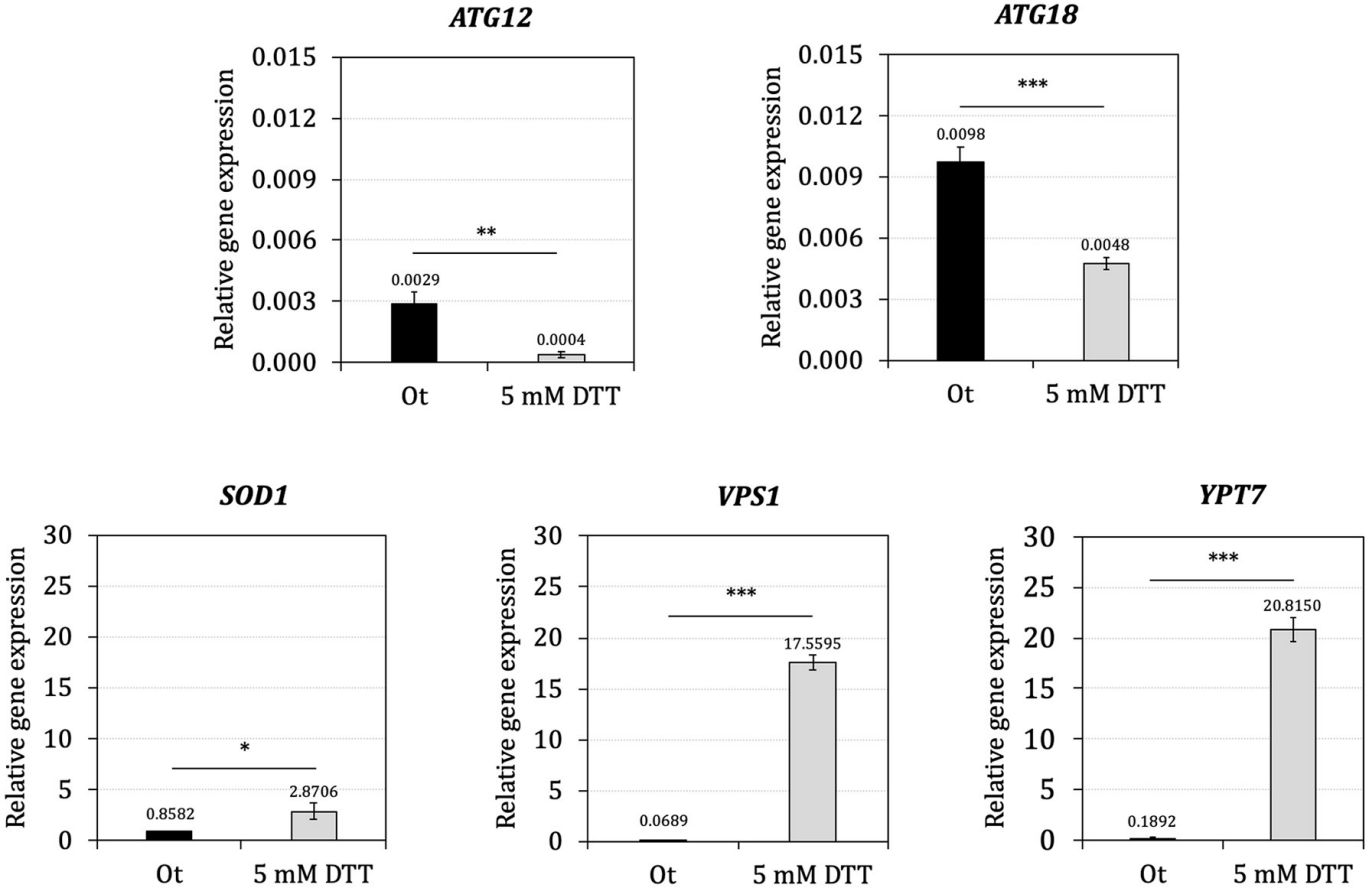
Transcriptional analysis of protein secretory related genes in wild-type *O*. *thermomethanolica* under ER stress. Data are shown as mean ± S.D. from three-independent biological replicate experiments (*n* = 3). *ACT* was used to normalize gene expression. Asterisks (*) indicate significant *p*-values as determined by independent sample *t*-test: *p<0.05; **p<0.01 and ***p<0.001.

Plasmids used in this study are described in Table 1. All yeast strains engineered in this study are derivatives of Ot-Mal-Xyl and Ot-Mal-Phy. For single gene mutagenesis, Ot-Cas9-Xyl, previously constructed by transformation of pOtAOX-Cas9 (Fig 1A in S1 File) into Ot-Mal-Xyl, was used as a transformation host. Briefly, pOtAOX-Cas9 was previously constructed by amplification of the Cas9 gene C-terminally tagged with SV40 nuclear localization signal from the vector p414-TEF1p-Cas9-CYC1t [22]. The amplified fragment was then joined with *Eco*RI/*Hin*dIII digested pOtAOX-Neo via Gibson Assembly (NEBuilder HiFi Assembly kit, New England Biolabs, UK) [25]. For modulation of gene expression, the Ot-dCas9-VP64-Xyl and Ot-dCas9-VP64-Phy strains were generated by transformation of pOtAOX-dCas9-VP64 (Fig 1B in S1 File) into both Ot-Mal-Xyl and Ot-Mal-Phy. The pOtAOX-dCas9-VP64 plasmid was constructed by amplification of the dCas9 gene N-terminally tagged with SV40 nuclear localization signal and C-terminally fused to four tandem copies of herpes simplex viral protein 16 (VP64) from pTPGI_dCas9_VP64 [29] using dCas9-F and dCas9-R primers (Table 2). The amplified DNA fragment was then joined with *Eco*RI/*Hin*dIII digested pPasNeo-OTOT [25] to yield pOtAOX-dCas9-VP64. The engineered yeast expressing dCas9-VP64 were verified by PCR amplification using the site-specific integration primers, Int-pAOX-F and Int-dCas9-VP64-R (Table 2).

**Fig 2:**
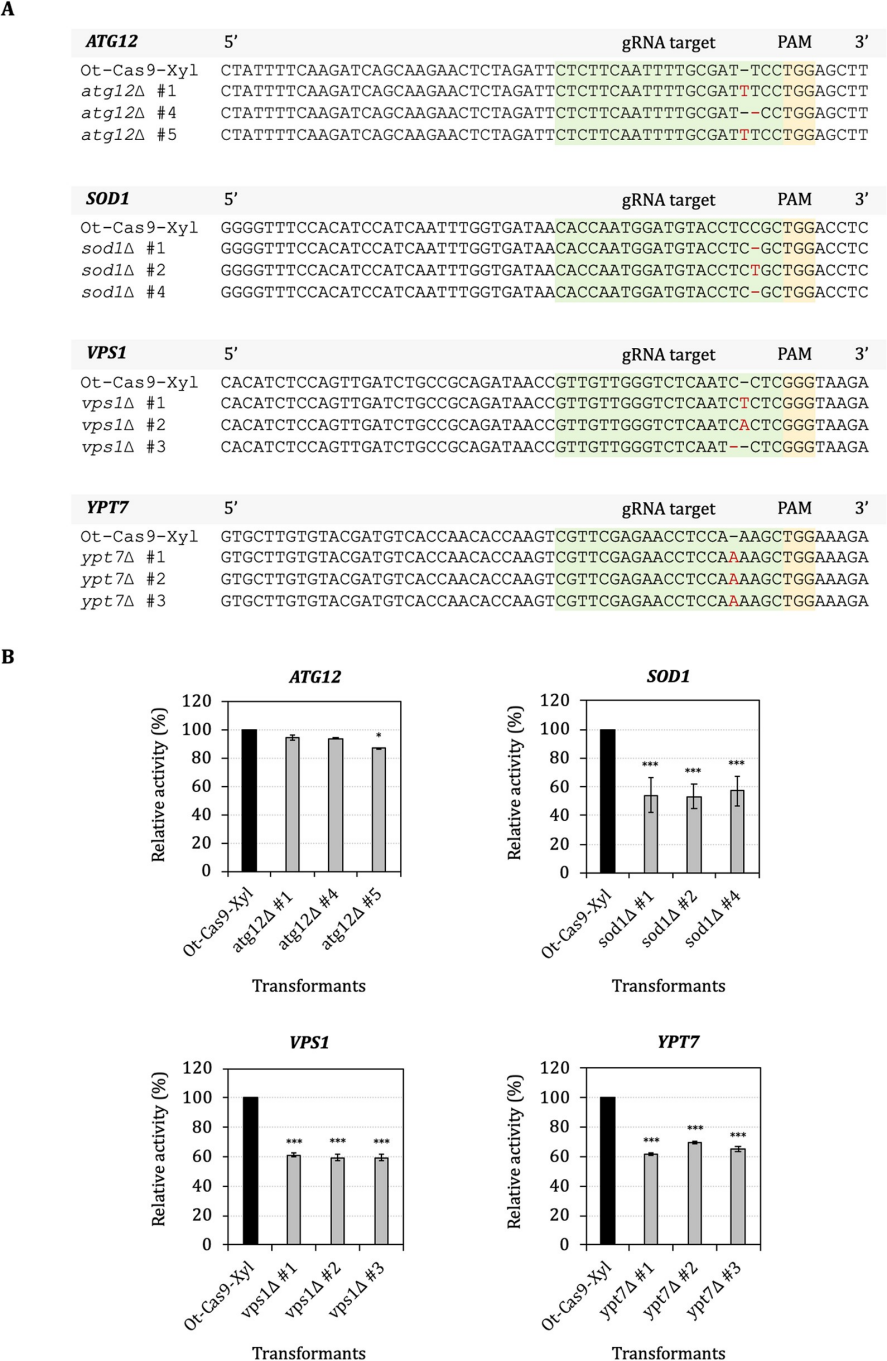
Mutagenesis of protein secretory related genes mediated by CRISPR-Cas9. Multiple sequence alignment of the protein secretory related genes of mutant strains. The modified regions from mutant strains are aligned with the Ot-Cas9-Xyl control. Red dash indicates deletion and red letter indicates insertion mutation (A). Relative activity of xylanase activity (%U/OD) of disrupted mutant yeasts compared with the Ot-Cas9-Xyl control (B). Data are shown as mean ± S.D. from three-independent biological replicate experiments (*n* = 3). Asterisks (*) indicate significantly altered expression profiles relative to Ot-Cas9-Xyl (*p<0.05 and ***p<0.001).

**Fig 3:**
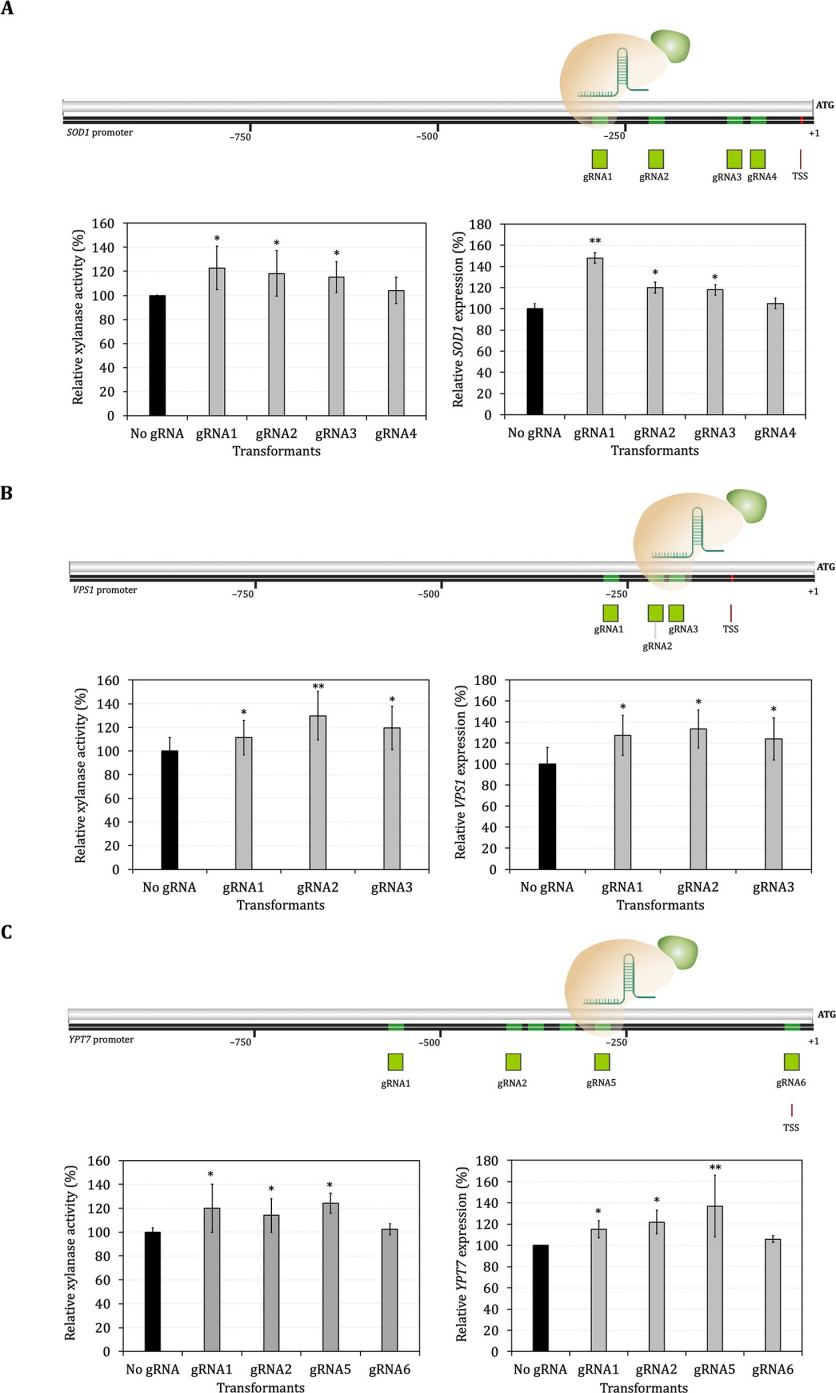
Targeting CRISPR-dCas9 to sequences upstream promoter of *SOD1* (A), *VPS1* (B) and *YPT7* (C) and relative activity of xylanase activity (%U/OD) and relative gene expression level (%fold change) in recombinant yeast harboring CRISPR-dCas9 system. Data are shown as mean ± S.D. from three-independent biological replicate experiments (*n* = 3). *ACT* was used to normalize gene expression. TSS is a putative transcription start site. Asterisks (*) indicate significantly altered expression profiles relative to the control Ot-dCas9-VP64 without gRNA (No gRNA) (*p<0.05 and **p<0.01).

The gRNA sequences for mutagenesis (Fig 2 in S1 File and Table 1 in S2 File) and activation of gene expression (Fig 3 in S1 File and Table 2 in S2 File) were designed using the CT-Finder program [34]. Eukaryotic transcription start sites (TSS) were predicted by a neural network promoter prediction online tool [35]. The self-cleaving ribozymes flanking the gRNA cassette (Hammerhead ribozyme-20 bp specific determinant sequence-structural gRNA-Hepatitis delta virus ribozyme, Fig 1C in S1 File) were designed following the protocol as described by Gao and Zhao [36] and synthesized by GenScript Biotech (Singapore) PTE. LTD. The pOtAOX-gRNA expression plasmids were constructed by insertion of the 231-bp gRNA cassette fragment into pOtAOX-Hyg via the *Eco*RI and *Kpn*I restriction sites. Constructed plasmids were verified by PCR using OtAOX-F and OtAOX-R primers and DNA sequencing (Macrogen, Korea) using the OtAOX-R primer (Table 2).

To obtain engineered yeast expressing dCas9-VP64, the *Mfe*I-linearized pOtAOX-dCas9-VP64 plasmid was introduced into both Ot-Mal-Xyl and Ot-Mal-Phy cells. For engineered yeast expressing gRNAs, *Bgl*II-linearized pOtAOX-gRNAs was either introduced into Ot-Cas9-Xyl, Ot-dCas9-VP64-Xyl or Ot-dCas9-VP64-Phy cells by electroporation according to a previously described protocol [3]. Transformants were then placed on YPD agar plate containing 100 μg mL^–1^ G418 for Ot-expressing dCas9-VP64 and 100 μg mL^–1^ hygromycin for Ot-expressing gRNAs. Plates were incubated at 30°C for 2–3 days until colonies were observed.

### SDS-PAGE and Western blot analysis of dCas9-VP64

Expression of dCas9-VP64 in Ot-expressing xylanase and phytase under pOtAOX was evaluated by SDS-PAGE and Western blot analysis. Proteins were extracted from overnight-cultivated cells. The cell suspension was mixed with 300 μL of 2 N NaOH and incubated at room temperature for 10 min. Cells were then centrifuged at 13,000 rpm for 2 min, resuspended in 200 μL of 1× SDS sample buffer and boiled for 10 min. Proteins were separated in sodium dodecyl sulfate (SDS) 12% Bis-Tris polyacrylamide gel at constant current (0.03 A) for 90 min. After SDS-PAGE, the separated proteins were transferred to a nitrocellulose membrane by wet-electroblotting (100 V for 90 min). The membrane was blocked with 5% (w/v) skim milk in TBST (6.05 g L^–1^ Tris-base, 8.76 g L^–1^ NaCl and 500 μL Tween 20; pH 7.6) overnight and then washed with TBST. The membrane was probed with monoclonal anti-Cas9 (7A9-3A3, Mouse mAb, #14697, NEB, MA, USA) (1:1,000 dilution) followed by actin antibody (C-2) (sc-8432, Santa Cruz Biotechnology, TX, USA) (1:1,000 dilution). Primary antibodies were detected with anti-Mouse IgG (whole molecule)-alkaline phosphatase (1:10,000 dilution) and 5-bromo-4-chloro-3-indolyl phosphate and *p*-nitroblue tetrazolium chloride (BCIP/NBT) substrate.

### Screening of engineered yeasts

Engineered yeast strains with single gene disruption or modulated expression were screened by PCR amplification of the hygromycin resistance gene (Hyg^R^) using Hyg-F and Hyg-R primers (Table 2). The expression of Cas9 and gRNA elements is controlled by derepression with glycerol under the pAOX promoter. For single gene disruption experiments, single colonies of Ot-Cas9-Xyl expressing gRNA were inoculated into YP broth supplemented with 2% glycerol (YP2G). A loop of cell suspension was streaked onto a YP2G plate and incubated at 30°C for 2–3 days until colonies were observed. Three single gene disrupted mutants were selected to identify the introduced mutation by PCR using specific primers for each target gene (Table 2). Consequently, verified transformants with either single gene disruption or modulated expression were used for enzyme quantification analysis.

### Enzyme expression and quantification

Heterologous xylanase and phytase were expressed by growing the transformants in 5 mL YPD supplemented with 100 μg mL^–1^ zeocin at 30°C, 250 rpm for 24 h. Subsequently, the inoculum was transferred to 5 mL of YPS (10 g L^–1^ yeast extract, 20 g L^–1^ peptone, and 20 g L^–1^ sucrose) at the initial OD_600_ at 0.2 and incubated at 30°C, 250 rpm for 24 h. Either Ot-Cas9-Xyl, Ot-dCas9-VP64-Xyl or Ot-dCas9-VP64-Phy without gRNA were used as controls. After cultivation, 1 mL samples of cell suspension were analyzed for cell growth by using SmartSpec Plus Spectrophotometer (BioRAD, USA) at OD_600_ nm and assayed for enzyme activity. The enzyme activity of fungal xylanase in culture supernatant towards 1% beechwood xylan (Sigma-Aldrich, MO, USA) was determined following a previously described method [37]. The xylanase activity was quantified by the 3,5-dinitrosalicyclic acid (DNS) method [38]. The activity was measured by spectrophotometer at OD_540_ nm. The phytase activity was determined by the color formation between molybdate and the released inorganic orthophosphate from phytate [39] following a previously described method [40]. The enzyme activity was measured by spectrophotometry at OD_405_ nm. One unit of enzyme activity was defined as the amount of enzyme that liberates 1 μ mole per min of either reducing sugar or inorganic phosphate. The enzymatic activities of different strains were normalized by cell density at OD_600_ nm (U/OD) for comparison of enzyme production from different cultures.

### Transcriptional analysis by quantitative real-time PCR

Total RNA was isolated from yeast cells using an RNeasy Mini Kit (Qiagen, Germany). 1 μg of total RNA was treated with DNase I (Thermo Fisher Scientific, MA, USA). RNA quality and quantity were verified by spectrophotometry at the absorbance of 260 and 280 nm using Nanodrop ND-1000 (Thermo Fisher Scientific, MA, USA). Two hundred nanograms of RNA were used as a template for first strand cDNA synthesis using a Revert Aid First Strand cDNA Synthesis Kit (Thermo Fisher Scientific, MA, USA). Quantitative real-time PCR analysis was performed using a Real-Time thermal cycler (Bio-Rad, CA, USA). Real-time PCR mixtures were prepared using the iQ^™^ SYBR Supermix (Bio-Rad, CA, USA). The reaction was carried out in a total volume of 10 μL containing 200 ng of cDNA, 2 μL of 1 μM of gene-specific forward and reverse primers (Table 2) and 5 μL of SYBR Green Supermix. Each sample was analyzed in technical triplicate and a no-template control for each pair of primers was included. The amplification conditions were 95°C for 4 min; 40 cycles of 95°C for 15 sec and the appropriate annealing temperature for 30 sec. At the end of the amplification step, a melting analysis was conducted to verify the specificity of the reaction by heating the amplified products from 55°C to 95°C with continuous fluorescent reading at 0.5°C increments. Gene expression level was described in terms of fold change compared with the strain harboring empty vector (No gRNA). Endogenous *O*. *thermomethanolica* actin (*ACT*) gene was used as an internal control and the gene expression level was calculated as previously described [41].

### Statistical analysis

Data were subjected to statistical analysis using either one-way ANOVA using post hoc correction by Duncan’s multiple range test or independent sample *t*-test (IBM Statistic SPSS, version 23). Data were presented as mean ± S.D. Post-hoc corrected *p*-values less than 0.05, 0.01 or 0.001 were considered significant.

## Results

### Transcriptional analysis of UPR target genes under ER stress

The UPR is an intracellular signaling system that is activated by unfolded proteins and activates expression of numerous target genes to restore protein homeostasis in the ER [42]. To test the effect of ER stress on UPR target genes expression, we examined transcriptional targets of this signaling pathway in *O*. *thermomethanolica* by monitoring mRNA levels of five genes involved in the secretory pathway (*ATG12*, *ATG18*, *SOD1*, *VPS1* and *YPT7*) under ER stress. Briefly, yeast was grown at mid-log phase (8 h) in YPD, then exposed with strong reducing agent 5 mM DTT for 60 min and determined the expression level by comparison to untreated control. Out of 5 selected genes, *SOD1*, *VPS1* and *YPT7* were strongly induced by DTT treatment, whereas the autophagy-related genes, *ATG12* and *ATG18* were repressed (Fig 1).

### Mutagenesis of genes involved in the secretory pathway mediated by CRISPR-Cas9

**Fig 4:**
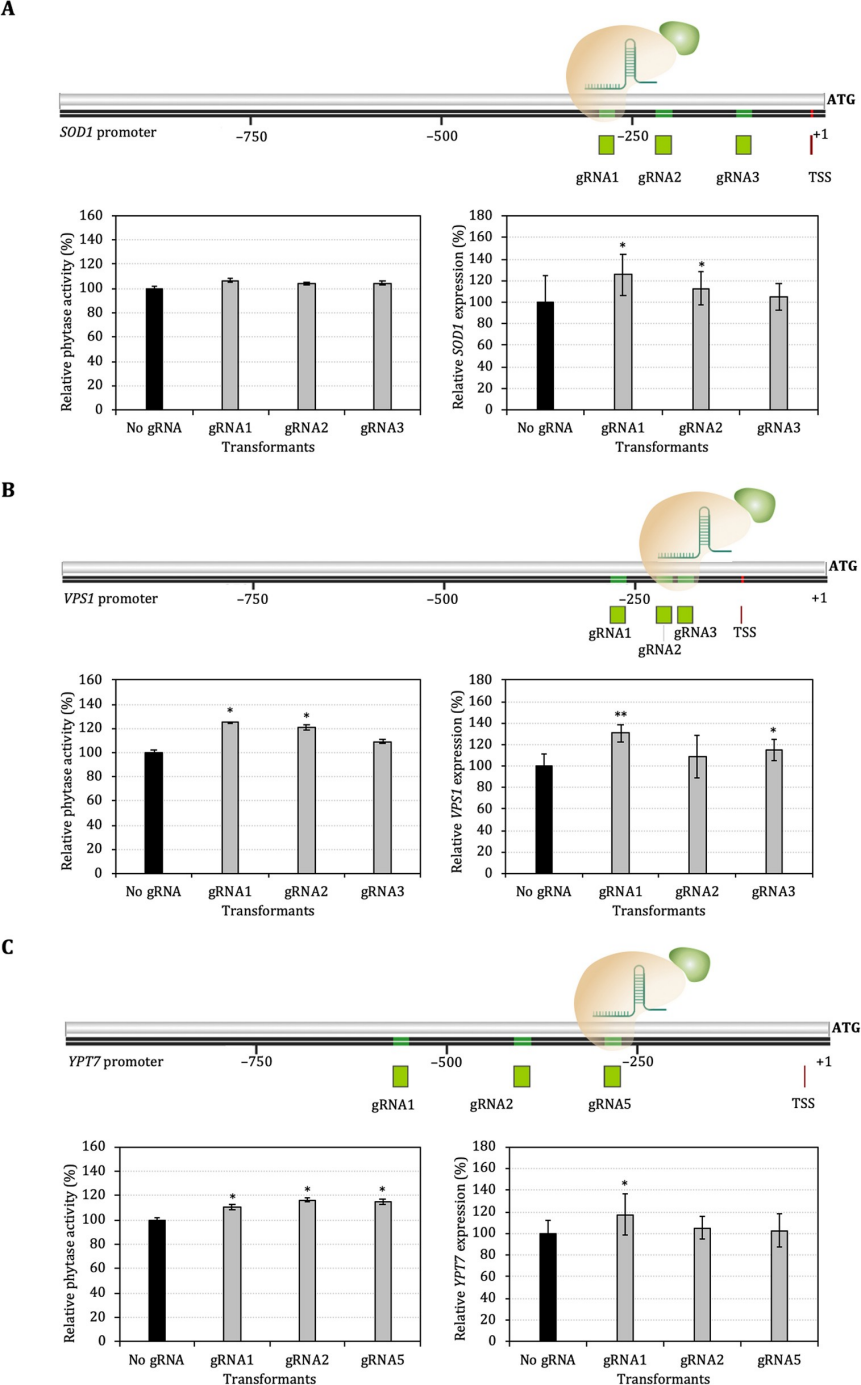
Targeting CRISPR-dCas9 to sequences upstream promoter of *SOD1* (A), *VPS1* (B) and *YPT7* (C) and relative activity of phytase activity (%U/OD) and relative gene expression level (%fold change) in recombinant yeast harboring CRISPR-dCas9 system. Data are shown as mean ± S.D. from three-independent biological replicate experiments (*n* = 3). *ACT* was used to normalize gene expression. TSS is a putative transcription start site. Asterisks (*) indicate significantly altered expression profiles relative to the control Ot-dCas9-VP64 without gRNA (No gRNA) (*p<0.05 and **p<0.01).

To investigate the function of proteins secretory-related genes, *ATG12*, *ATG18*, *SOD1*, *VPS1* and *YPT7* genes were selected for CRISPR-Cas9 mediated mutagenesis and examined their impact on secretion of xylanase model protein using DNS method. gRNA sequences targeting each gene were designed (Fig 2 in S1 File and Table 1 in S2 File) and cloned into pOtAOX-Hyg for expression of gRNAs. Ot-Cas9 expressing xylanase host was transformed with plasmids to obtain recombinant yeast expressing gRNAs. CRISPR-Cas9 induced point mutations generating premature stop codons were identified in *ATG12*, *SOD1*, *VPS1* and *YPT7* genes and thus are likely to result in loss of protein function (Fig 2A, Fig 4 in S1 File). However, no mutations were found for the *ATG18* gene (data not shown). Then, three selected clones for each single gene disrupted mutants were selected to analyze the xylanase activity in order to demonstrate the effect of those genes on protein secretion. In this work, reduction of xylanase activity was found in *sod1*Δ (45.22%), *vps1*Δ (39.98%) and *ypt7*Δ (34.54%) mutants compared with control, whereas xylanase activity was unchanged in the *atg12*Δ mutant (Fig 2B and Table 3 in S2 File).

### Growth profiling and expression of dCas9-VP64 in recombinant yeasts

Based on the observation that loss of function mutants for the *SOD1*, *VPS1* and *YPT7* genes showed significantly decreased secretion of xylanase, we hypothesized that activation of these genes using CRISPRa that specific to target sequences could lead to increased heterologous xylanase and phytase secretion. Therefore, recombinant yeast expressing dCas9-VP64 were constructed by transformation of pOtAOX-dCas9-VP64 into both Ot-Mal-Xyl and Ot-Mal-Phy strains. Site-specific integration of dCas9 at the native AOX promoter was verified by integration-specific PCR (S1 Fig) and expression of dCas9-VP64 protein (173 kDa) in transformants was verified by Western blotting (S2 Fig). Then, the effect of dCas9-VP64 expression on growth was tested by culturing transformants in YPD for 48 h, which showed no difference compared with the control (Fig 5A and 5C in S1 File). Moreover, the xylanase and phytase activities of all transformants were not significantly different from Ot-Mal-Xyl and Ot-Mal-Phy (Fig 5B and 5D in S1 File) under the same culture conditions. Ot-dCas9-VP64-Xyl and Ot-dCas9-VP64-Phy were consequently transformed with plasmids for expression of pOtAOX-gRNAs in CRISPRa experiments.

### Enhancement of xylanase and phytase secretion mediated by CRISPRa

Multiple gRNA sequences targeting the putative promoter regions upstream of *SOD1*, *VPS1* and *YPT7* genes were designed (Fig 3 in S1 File and Table 2 in S2 File) and cloned into pOtAOX-Hyg plasmid for expression of pOtAOX-gRNAs and subsequently transformed into Ot-dCas9-VP64-Xyl and Ot-dCas9-VP64-Phy. In order to test for the CRISPRa effect on protein secretory-related genes, the enzyme activity of transformants expressing gRNAs was compared with that of the control strain harboring empty vector (No gRNA).

The expression of gRNAs targeting different regions upstream of the *SOD1*, *VPS1* and *YPT7* genes resulted in significantly higher levels of xylanase activity compared with the control (No gRNA), although the effect was small and variable among different target genes and gRNAs (Fig 3A–3C and Table 4 in S2 File). For example, xylanase activity was generally increased by CRISPRa targeting of the *SOD1* promoter, however, the increase ranged from 4.06% in case of gRNA4 to 22.84% for gRNA1 (Fig 3A and Table 4 in S2 File). We also tested whether CRISPRa of protein secretory-related genes could affect the secretion of a different heterologous protein (glycosylated phytase). Three different gRNAs targeting the regions upstream of *SOD1* (gRNA1, gRNA2 and gRNA3), *VPS1* (gRNA1, gRNA2 and gRNA3) and *YPT7* (gRNA1, gRNA2 and gRNA5) were selected and Ot-dCas9-VP64-Phy was transformed with the plasmids for expression of these gRNAs. It was found that higher heterologous phytase activity was also observed in strains expressing gRNAs targeting the regions upstream of the *SOD1* (6.69, 4.19 and 4.59% of gRNA1, 2 and 3, respectively), *VPS1* (25.39, 20.63 and 9.13% of gRNA1, 2 and 3, respectively) and *YPT7* (10.56, 16.14 and 14.79% of gRNA1, 2 and 5, respectively) genes (Fig 4 and Table 6 in S2 File). Furthermore, the CRISPRa effect of expressing gRNAs targeting promoter regions was verified by measuring the expression level of target genes via quantitative real-time PCR (RT-qPCR). The RT-qPCR analysis revealed that, consistent with increment of heterologous protein secretion, the expression levels of *SOD1*, *VPS1 and YPT7* genes were significantly upregulated (Figs 3 and 4 and Tables 5 and 7 in S2 File).

## Discussion

The UPR regulates gene expression in response to ER stress by allowing the cell to tolerate folding stress [43]. However, transcriptional changes associated with the UPR have not been characterized in *O*. *thermomethanolica*. Therefore, transcriptional analysis of five candidate UPR target genes were firstly monitored under ER stress by treating cells with the potent ER stressor (DTT) that disrupts protein folding by preventing disulfide bond formation [43]. Of the five genes tested, *SOD1*, *VPS1*, and *YPT7* showed strong upregulation, whereas *ATG12* and *ATG18* were downregulated. These results suggest that *SOD1*, *VPS1* and *YPT7* genes are UPR target genes whereas *ATG12* and *ATG18* genes may be associated with other protein secretory pathways rather than UPR. Furthermore, mutants with disruptions of *ATG12*, *SOD1*, *VPS1* and *YPT7* genes were obtained, whereas no mutant could be isolated for *ATG18* suggesting that it is essential, or the gRNA targeting this gene is ineffective for recruiting Cas9. Additionally, xylanase activity was significantly reduced in *sod1*Δ, *vps1*Δ and *ypt7*Δ mutants compared with the control strain. However, xylanase activity was not significantly altered in the *atg12*Δ mutant.

The cytoplasm-to-vacuole targeting (Cvt) or autophagy-related pathway is another pathway of vacuole protein transport from the cytosol to the vacuole [44]. The Cvt pathway is reported to overlap extensively with selective macroautophagy that has been explored in the methylotrophic yeasts *Pichia pastoris* and *O*. *polymorpha* [45]. Macroautophagy is activated by ER stress as a cellular survival mechanism. Ubiquitin-like protein conjugation complexes are formed by the autophagy (Atg) proteins, Atg5, Atg12 and Atg16. Although Atg12 is assumed to play an important role in autophagy, which is a conserved degradative transport pathway [46], similarly to our result, Marsalek *et al*. reported that disruption of the autophagy-related Cvt pathway genes *ATG8* and *ATG11* did not affect the secretion of heterologous proteins in *P*. *pastoris* [47].

In the methylotrophic yeast, Ypt7 is also reported to be involved in the Cvt pathway of *P*. *pastoris* [48]. A recent report found that deletion of *YPT7* increased secretion of the model proteins HyHEL-Fab and CES in the yeast *P*. *pastoris* [47], and of model proteins cellulase (Ctcel8A) and exocellulase (PCX) in *S*. *cerevisiae* [49,50]. Nevertheless, loss of either *YPT7* or *CCZ1* (*YPT7*-homolog) functions result in a highly aberrant vacuolar morphology and disruption of three main transport pathways to the vacuole (endocytosis, carboxypeptidase Y and alkaline phosphatase) in *S*. *cerevisiae* [48,51,52]. Hence, the reduction of xylanase activity observed in the *ypt7*Δ mutant (Fig 2B) suggests a different role of Ypt7 in *O*. *thermomethanolica* compared with other yeasts.

Vps1 is a dynamin-like protein that is involved in several membrane fusion and fission events within the endosome, Golgi, and peroxisome [53], and also has an important role in pre-vacuolar secretion in *S*. *cerevisiae* [54]. Hence, the observed reduction of xylanase protein secretion in *vps1*Δ mutant (Fig 2B) might be the result of a defect in pre-vacuolar secretion as in other yeasts. These findings are in agreement with a previous study which showed that *vps1* deletion in *Candida albicans* results in filamentation defects and reduced secretion of extracellular protease [17]. Furthermore, a recent study showing that *vps1*Δ mutants are also sensitive to reactive oxygen species (ROS) caused by paraquat in fission yeast *Schizosaccharomyces pombe* [20].

High loading of unfolded heterologous proteins to the ER and the resulting ER stress leads to ROS production [55]. *S*. *cerevisiae sod1*Δ mutants are reported to sensitive even to atmospheric levels of oxygen and exhibit vacuolar fragmentation [56]. Therefore, reduction of protein secretion in *sod1Δ* in this study (Fig 2B) might be a consequence of high accumulated ROS in the ER which inhibits protein transport from the ER to the target destinations.

In this work, we next sought to develop a CRISPRa system in *O*. *thermomethanolica* for activation of genes of interest. Recombinant yeast expressing dCas9-VP64 were firstly constructed by transformation of pOtAOX-dCas9-VP64 into both Ot-Mal-Xyl and Ot-Mal-Phy strains. The constitutive expression of dCas9-VP64 in the absence of gRNA did not show reduction of growth or heterologous enzyme activity (Fig 5 in S1 File), suggesting that dCas9-VP64 has no confounding effect on growth, protein expression or secretion. The expression of gRNAs targeting different regions upstream of targeted genes resulted in various levels of increased target gene expression and enzymes activity, for example, targeting gRNA 1 in the *SOD1* promoter of Ot-dcas9-VP64 expressing phytase and xylanase increased *SOD1* gene expression up to 25.27% and 48.15%, respectively suggesting a significant CRISPRa effect. However, the CRISPRa effect was rather modest, with a less than 30% increase observed among all gRNA tested. Smith *et al*. documented that the efficacy of gRNA for recruiting dCas9 transcriptional regulators and mediating CRISPRa depends on the accessibility and location of the target region, in which gRNA targeting region between the –200 and +1 nucleotide relative to the transcription start site (TSS) are usually the most effective [57]. By using multiple gRNAs in the proximity of TSS, dCas9-VP64 has a strong CRISPRa effect in human HEK293 and HeLa cells and *S*. *cerevisiae* [58,59]. We designed gRNAs targeting regions assumed to be upstream of TSS and thus effective for CRISPRa, although other regions may be more effective.

Targeting of gRNAs to the *SOD1* upstream region were shown to mediate CRISPRa with increased *SOD1* gene expression and xylanase production. Increased expression of ROS-scavenger Sod1 could allow the yeast to tolerate the oxidative stress caused by unfolded protein in the ER, similar to that observed in yeast *K*. *lactis* where overexpression of *SOD1* was noted to increase human serum albumin (HSA) and glucoamylase production together with lower ROS [21]. In this study, the activation of *VPS1* and *YPT7* by CRISPRa also enhanced heterologous protein secretion. Vps1 and Ypt7 are involved in protein transport to vacuole and are also implicated in the fusion of the interacting membranes during the trafficking of cargo to the vacuole [18,60]. Therefore, activation of these vacuolar sorting-related genes could effectively reduce missorting of heterologous proteins in the Golgi-to-vacuole pathway and thus enhance secretion of heterologous protein.

## Conclusions

To evaluate the role of genes on heterologous protein secretion in the yeast *O*. *thermomethanolica* TBRC 656, we employed genetic tools to create mutants and performed phenotypic analyses. Mutants with disruptions of the *ATG12*, *SOD1*, *VPS1* and *YPT7* genes were obtained by CRISPR-Cas9 mutagenesis. Disruption of *SOD1*, *VPS1* and *YPT7* led to decreased expression of heterologous fungal xylanase activity, indicating that these genes could play important roles in controlling protein secretion. Activation of *SOD1*, *VPS1* and *YPT7* genes by CRISPRa led to significant activation of xylanase and phytase protein secretion, providing further evidence for the role of *SOD1*, *VPS1* and *YPT7* genes in regulating of protein secretion.

## Supporting information

S1 Fig Site-specific integration of dCas9-VP64. An agarose gel electrophoresis of 1,013 bp at the OtAOX promoter of (A) Ot-dCas9-VP64-Xyl and (B) Ot-dCas9-VP64-Phy. Lane M is 1 kb DNA ladder, no. 1–17 is transformants.
(DOCX)

S2 Fig Western blot analysis. dCas9-VP64 expressed band (173 kDa) of (A) Ot-dCas9-VP64-Xyl and (B) Ot-dCas9-VP64-Phy transformants. Either *O*. *thermomethanolica* expressing xylanase (Ot-Mal-Xyl) or phytase (Ot-Mal-Phy) used as a negative control (N) under YPS condition. The protein probed by monoclonal anti-Cas9 and anti-actin. Lane M, pre-stained protein molecular marker; purified Cas9 was loaded as positive control (P).
(DOCX)

S1 File Supplementary figures. (DOCX)

S2 File Supplementary tables. (DOCX)

S1 Raw images (PDF)

S2 Raw images (PDF)
