# Supplementary material for: Modulation of heterologous protein secretion in the thermotolerant methylotrophic yeast Ogataea thermomethanolica TBRC 656 by CRISPR-Cas9 system
Source: PLoS One. 2021 Sep 28;16(9):e0258005. doi: 10.1371/journal.pone.0258005 (PMC8478189; doi:10.1371/journal.pone.0258005)
Supplement: S1 Fig — An agarose gel electrophoresis of 1,013 bp at the OtAOX promoter of (A) Ot-dCas9-VP64-Xyl and (B) Ot-dCas9-VP64-Phy. Lane M is 1 kb DNA ladder, no. 1–17 is transformants. (DOCX) [file pone.0258005.s001.docx]

**
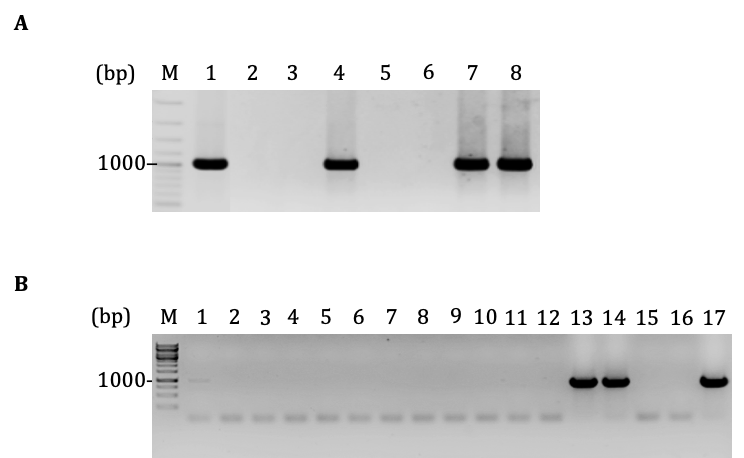
**

**S1 Fig. Site-specific integration of dCas9-VP64.** An agarose gel electrophoresis of 1,013 bp at the OtAOX promoter of (A) Ot-dCas9-VP64-Xyl and (B) Ot-dCas9-VP64-Phy. Lane M is 1 kb DNA ladder, no. 1-17 is transformants.
