# Supplementary material for: Modulation of heterologous protein secretion in the thermotolerant methylotrophic yeast Ogataea thermomethanolica TBRC 656 by CRISPR-Cas9 system
Source: PLoS One. 2021 Sep 28;16(9):e0258005. doi: 10.1371/journal.pone.0258005 (PMC8478189; doi:10.1371/journal.pone.0258005)
Supplement: S1 File — (DOCX) [file pone.0258005.s003.docx]

**Supporting Information**

**Modulation of heterologous protein secretion in the thermotolerant methylotrophic yeast *Ogataea thermomethanolica* TBRC 656 by CRISPR-Cas9 system**

Worarat Kruasuwan^1^, Aekkachai Puseenam^1^, Chitwadee Phithakrotchanakoon^2^,

Sutipa Tanapongpipat^1^ and Niran Roongsawang^1,^*

^1^ Microbial Cell Factory Research Team, Microbial Biotechnology and Biochemicals Research Unit, National Center for Genetic Engineering and Biotechnology, National Science and Technology Development Agency, 113 Thailand Science Park, Phahonyothin Road, Khlong Nueng, Khlong Luang, Pathum Thani 12120, Thailand

^2^ Microbial Systems and Computational Biology Research Team, Thailand Bioresource Research Center, National Center for Genetic Engineering and Biotechnology, National Science and Technology Development Agency, 113 Thailand Science Park, Phahonyothin Road, Khlong Nueng, Khlong Luang, Pathum Thani 12120, Thailand.

***Correspondence author**:

Niran Roongsawang, Ph.D. (NR)

Microbial Cell Factory Research Team, Microbial Biotechnology and Biochemicals Research Unit, National Center for Genetic Engineering and Biotechnology, National Science and Technology Development Agency, 113 Thailand Science Park, Phahonyothin Road, Khlong Nueng, Khlong Luang, Pathum Thani 12120, Thailand.

Tel. +66 2564 6700; Fax. +66 2564 6707

Email: niran.roo@biotec.or.th

**
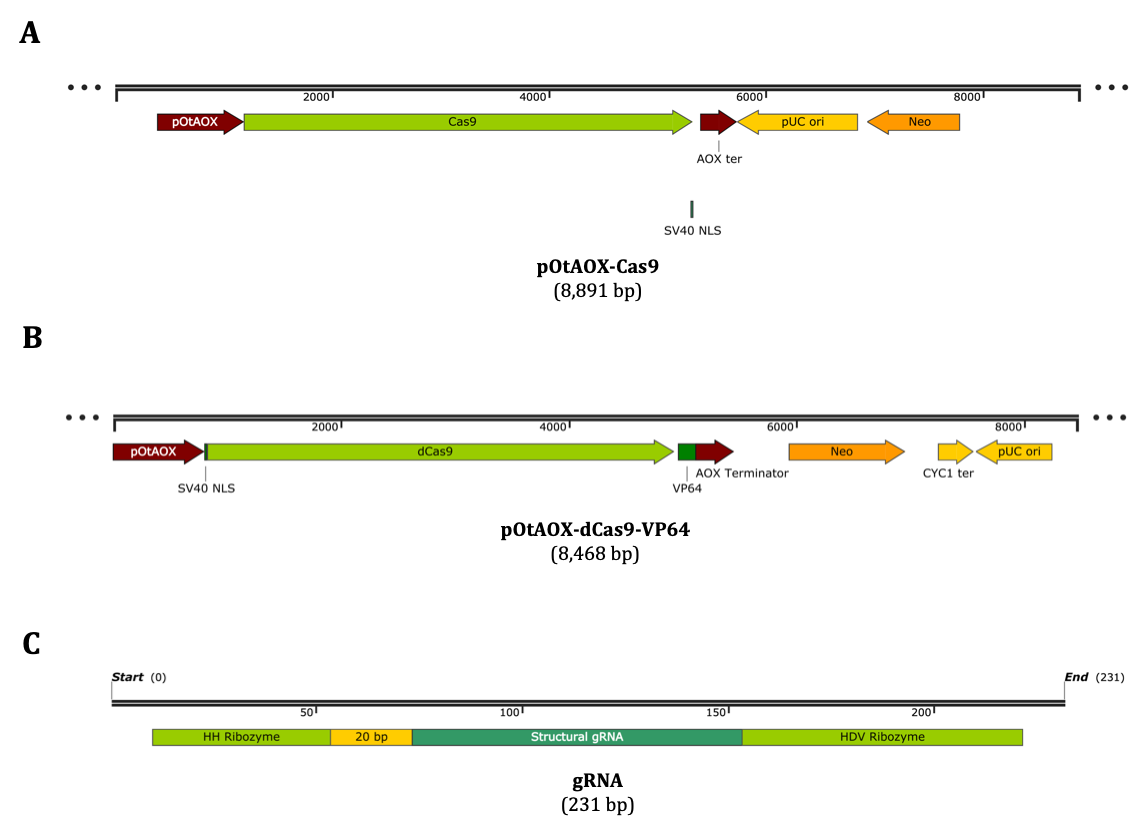
**

**Figure 1 Schematic view of the programmable CRISPR-Cas9 systems used in this study.** (A) pOtAOX-Cas9, (B) pOtAOX-dCas9-VP64 and (C) gRNA flanked with 2 self-cleaving ribozymes (HH and HDV ribozymes).

**A**


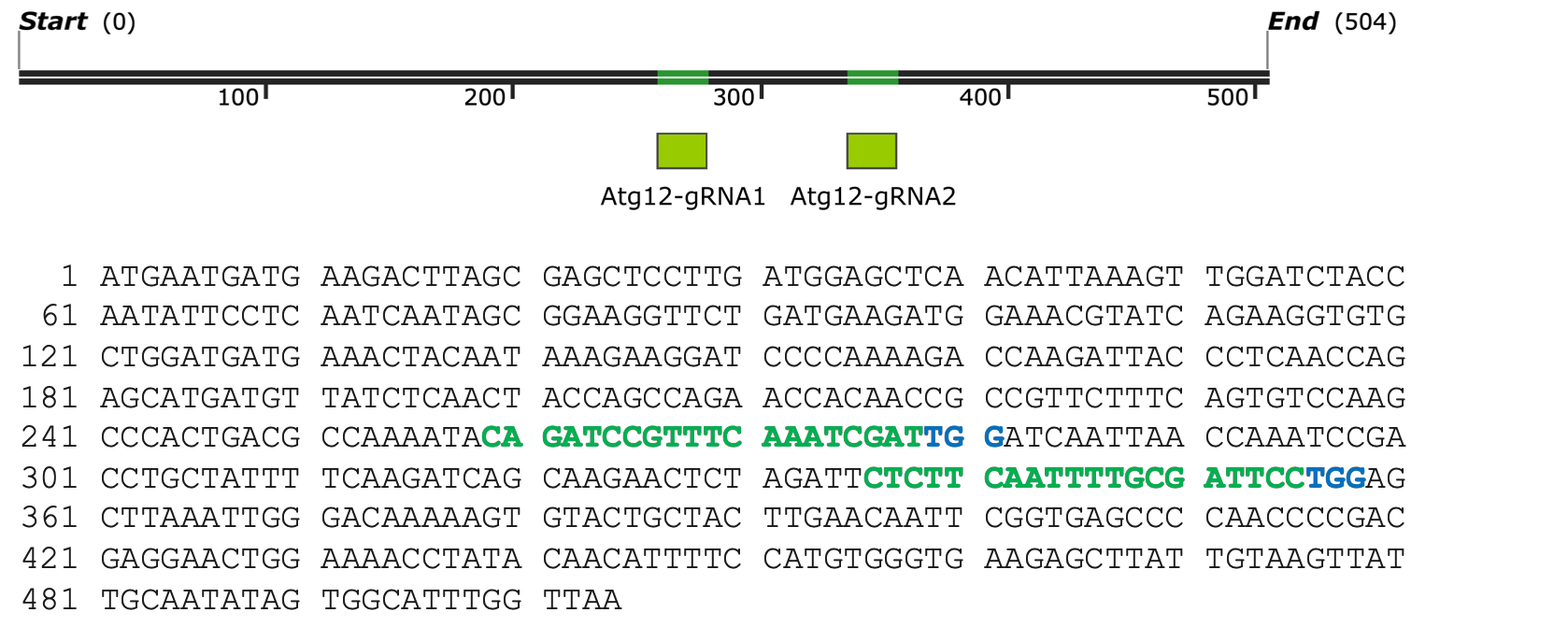


**B**

**
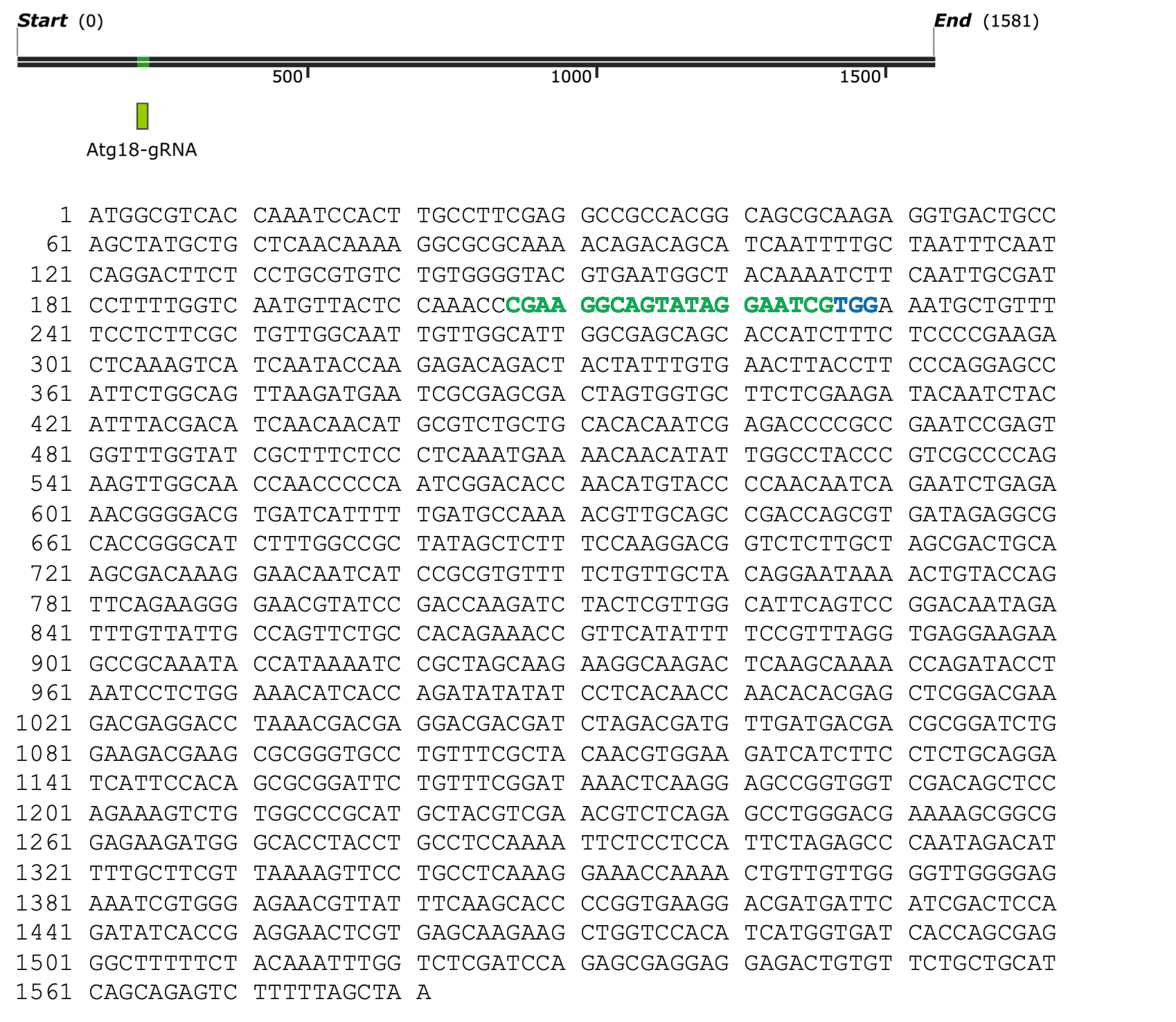
**

**C**

**
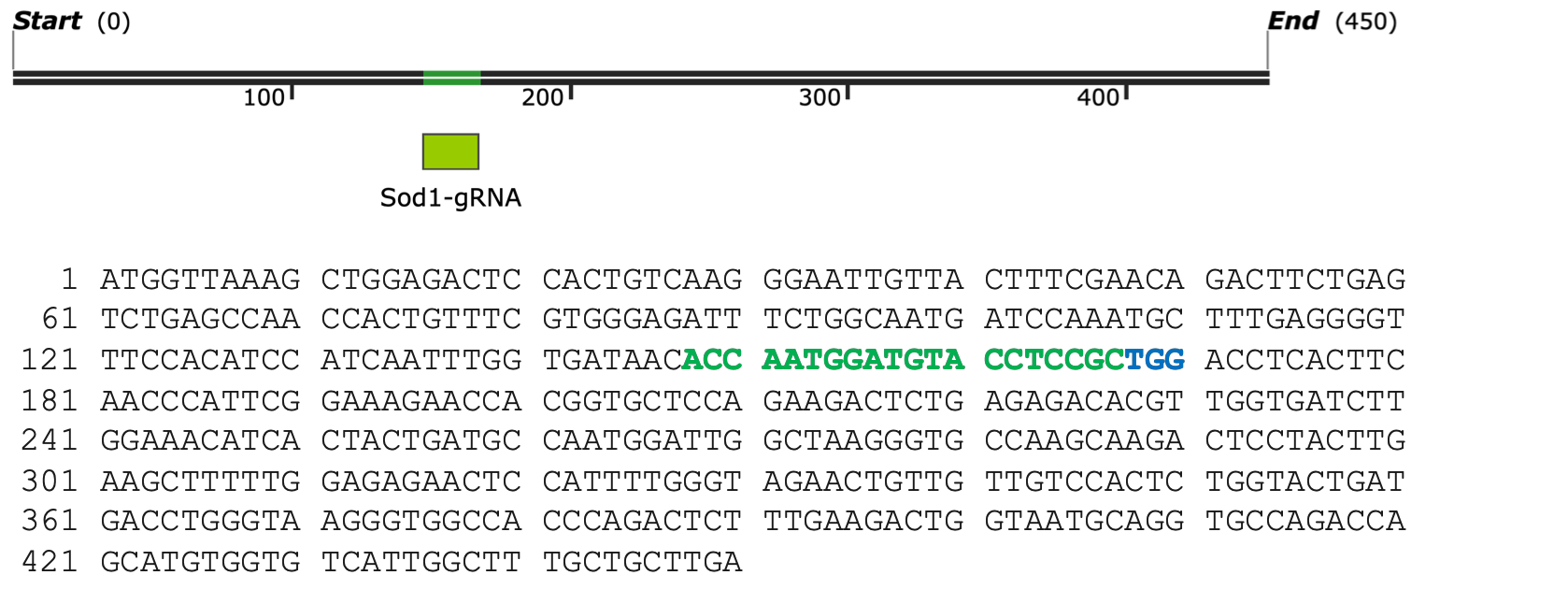
**

**D**

**
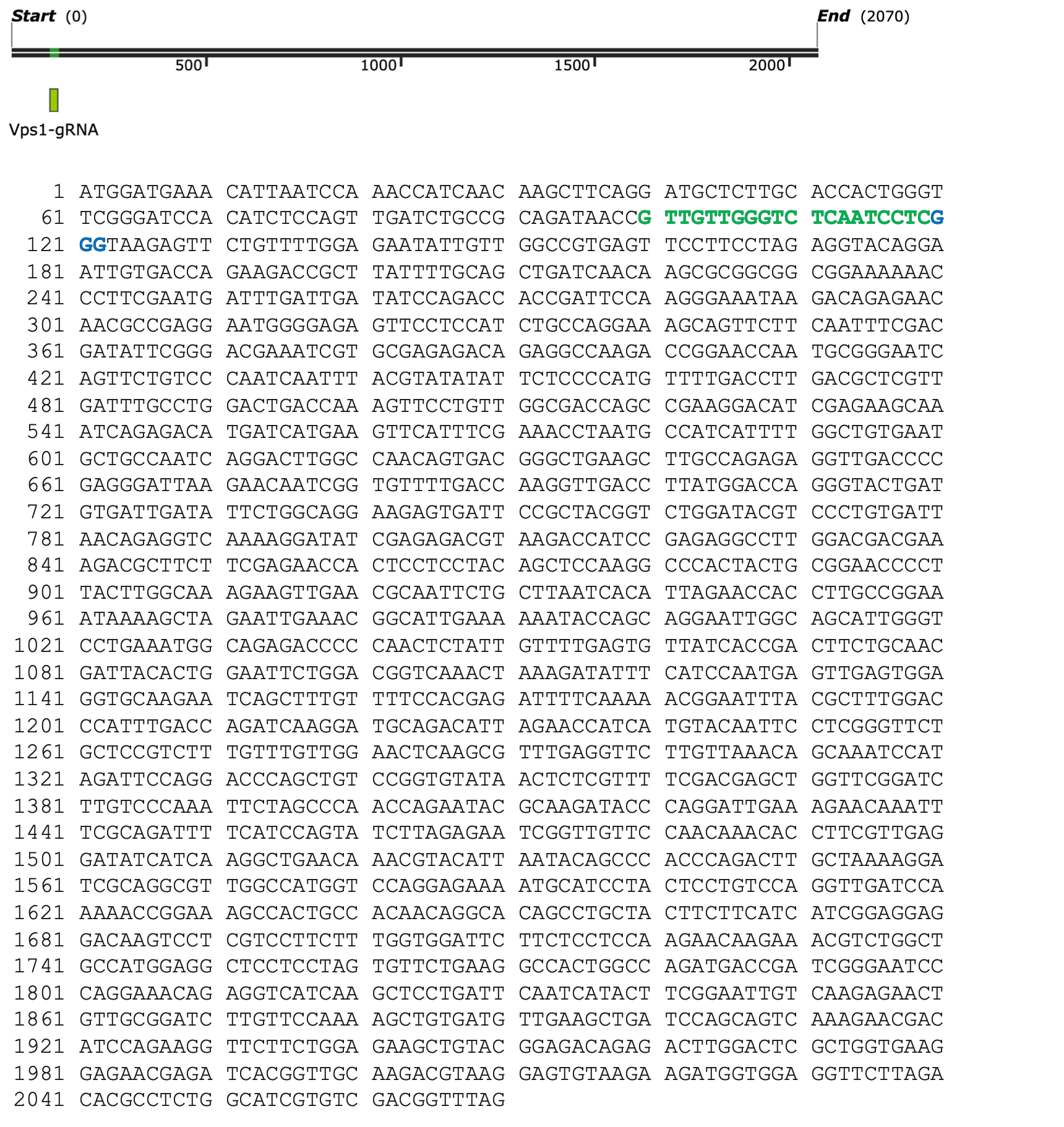
**

**E**

**
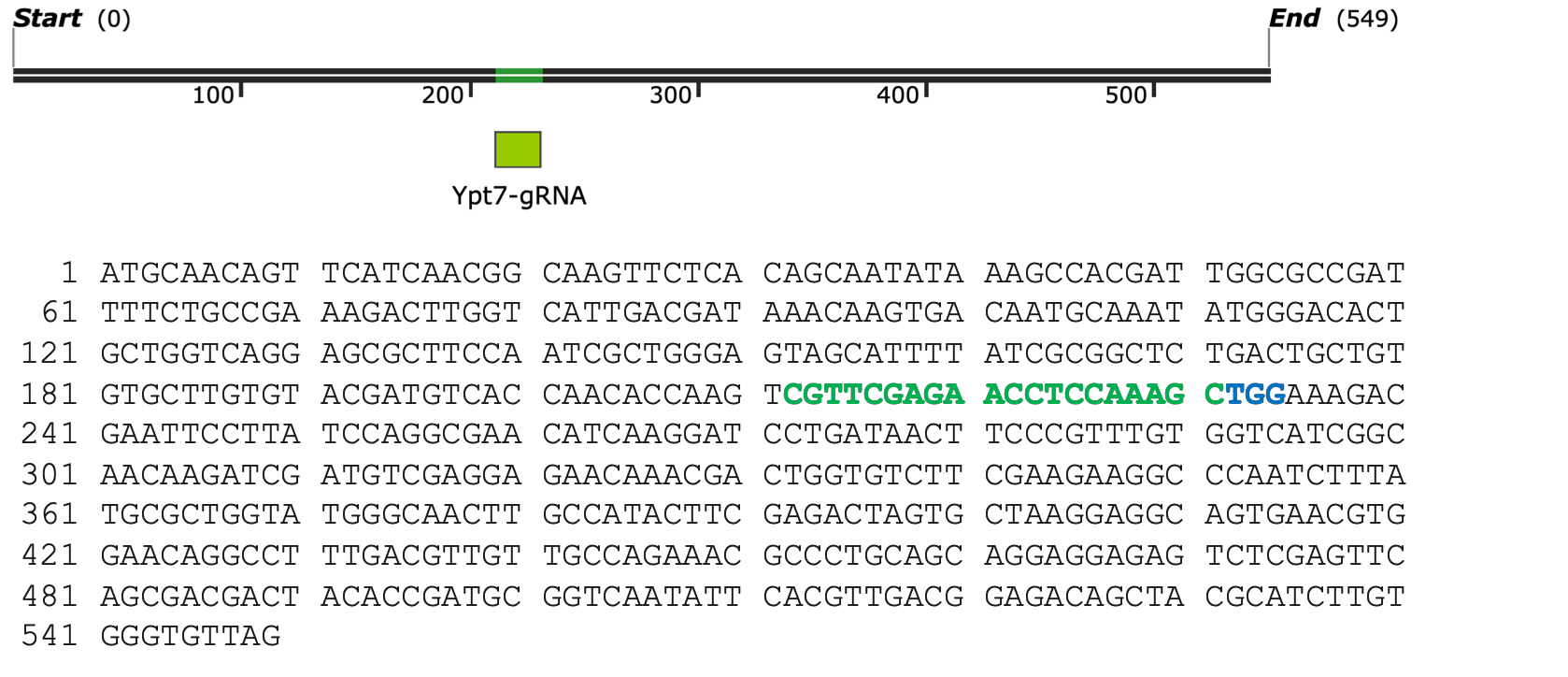
**

**Figure 2 Schematic view of nucleotide sequences and gRNA position of putative genes used in this study.** (A) ubiquitin-like protein ATG12 (*ATG12*), (B) autophagy-related protein 18 (*ATG18*), (C) superoxide dismutase (*SOD1*), (D) vacuolar protein sorting-associated protein 1 (*VPS1*) and (E) Rab GTP-binding protein YPT7 (*YPT7*) of *O. thermomethanolica*. The 20-bp specific determinant sequences of gRNA and PAM sequence are respectively in green and blue.

**A**

**
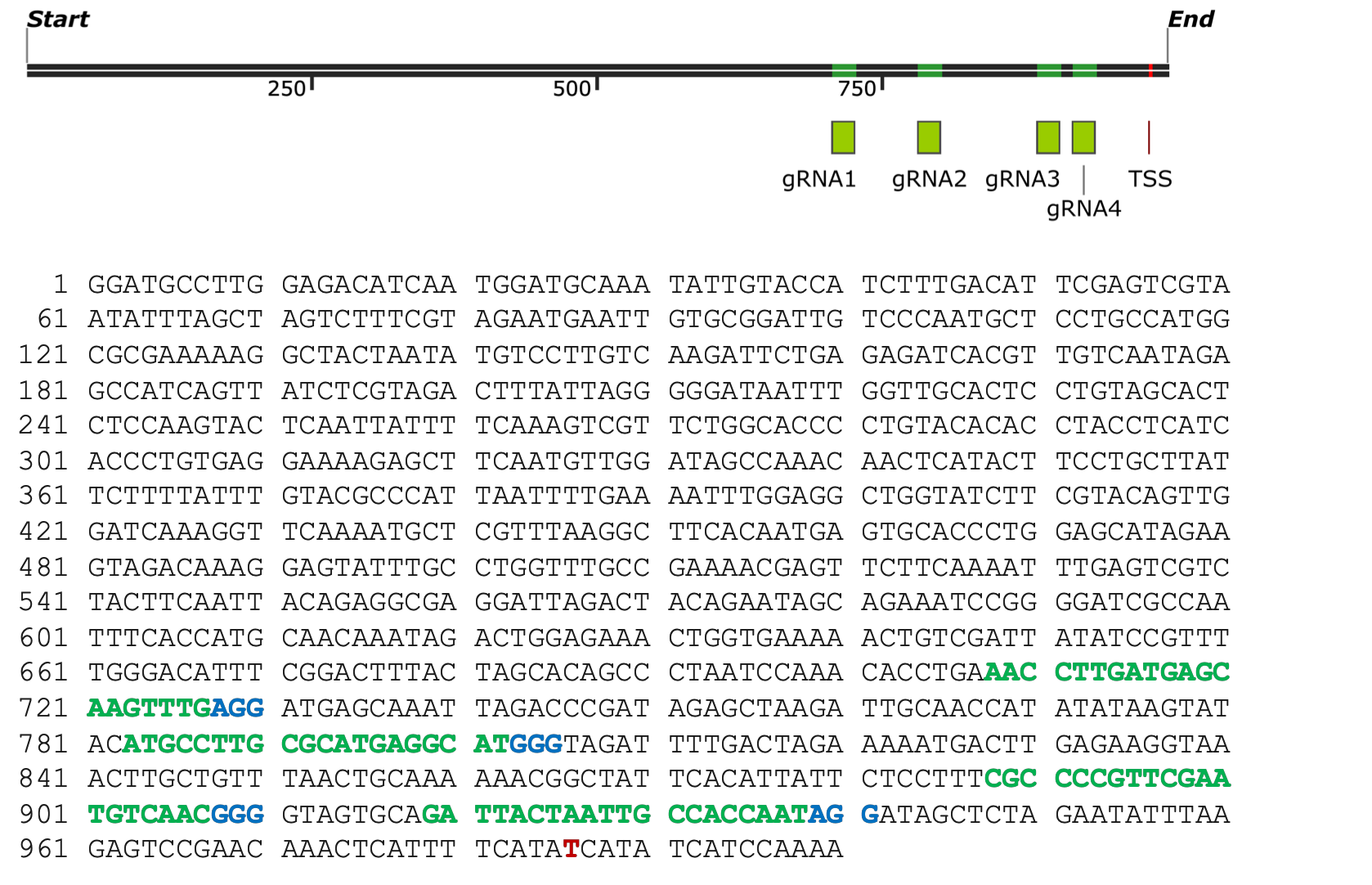
**

**B**

**
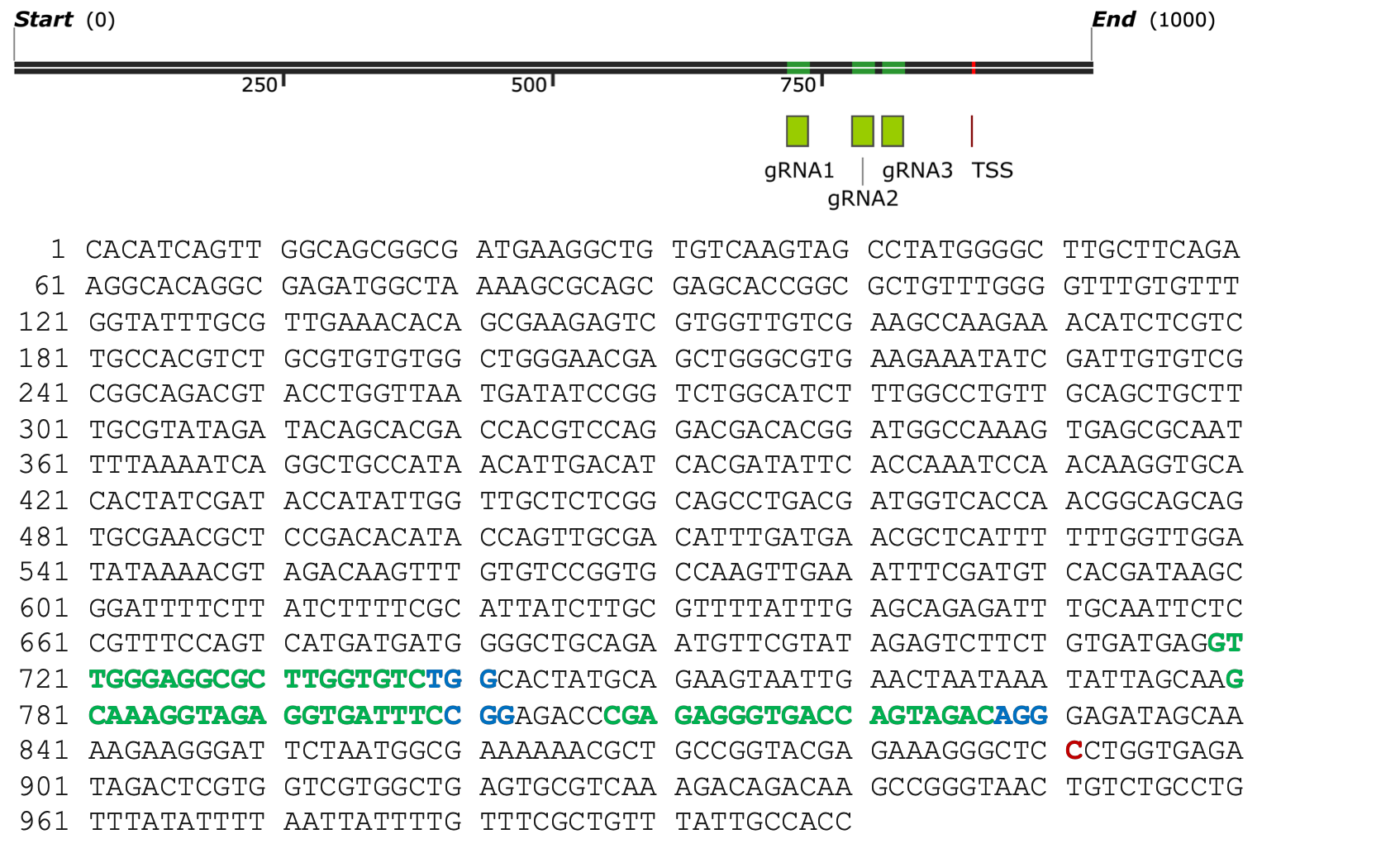
**

**C**

**
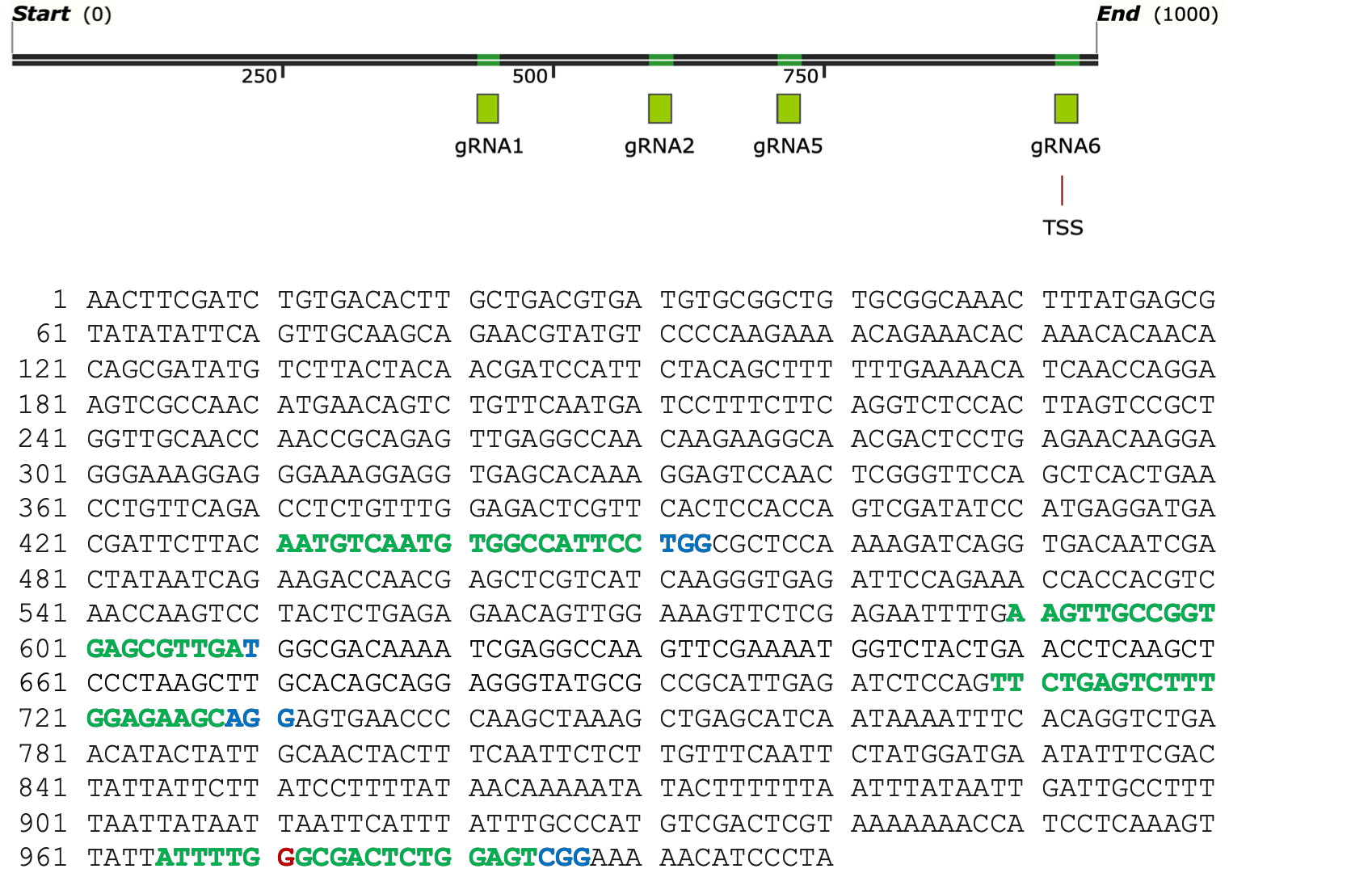
**

**Figure 3 Schematic view of nucleotide sequences and gRNA positions of upstream promoter of putative genes used in this study.** (A) superoxide dismutase (*SOD1*), (B) vacuolar protein sorting-associated protein 1 (*VPS1*) and (C) Rab GTP-binding protein YPT7 (*YPT7*) genes of *O. thermomethanolica*. The 20-bp specific determinant sequences of gRNA, PAM sequence and transcription start site (TSS) are respectively in green, blue and red.

**A**

**B**


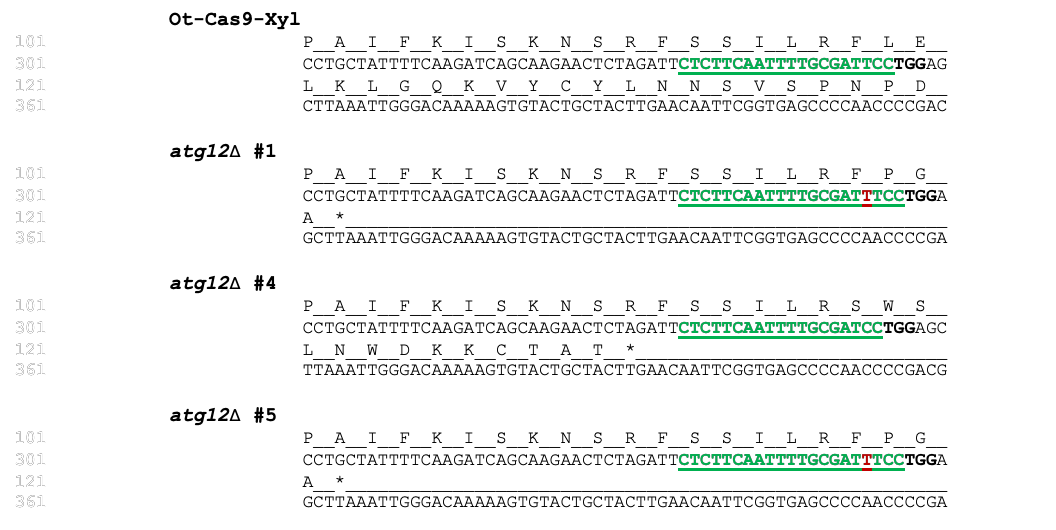

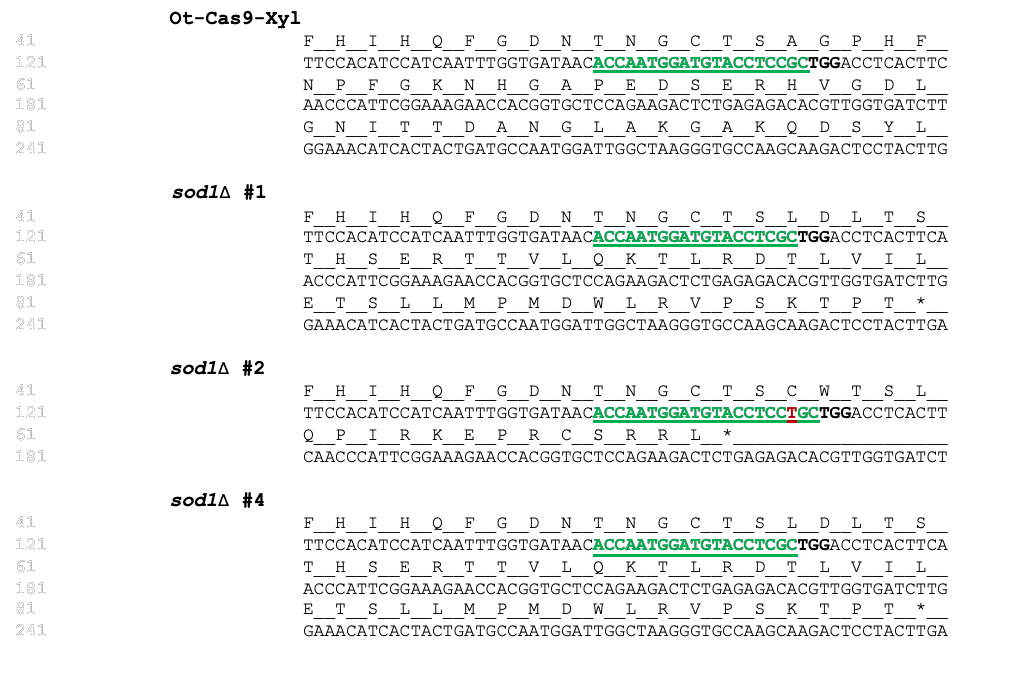


**C**

**D**


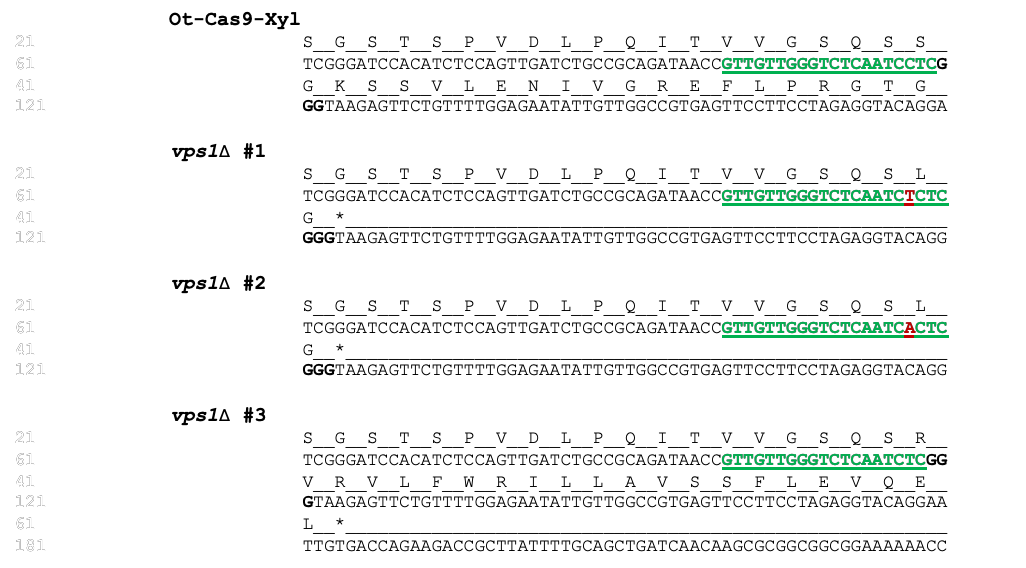

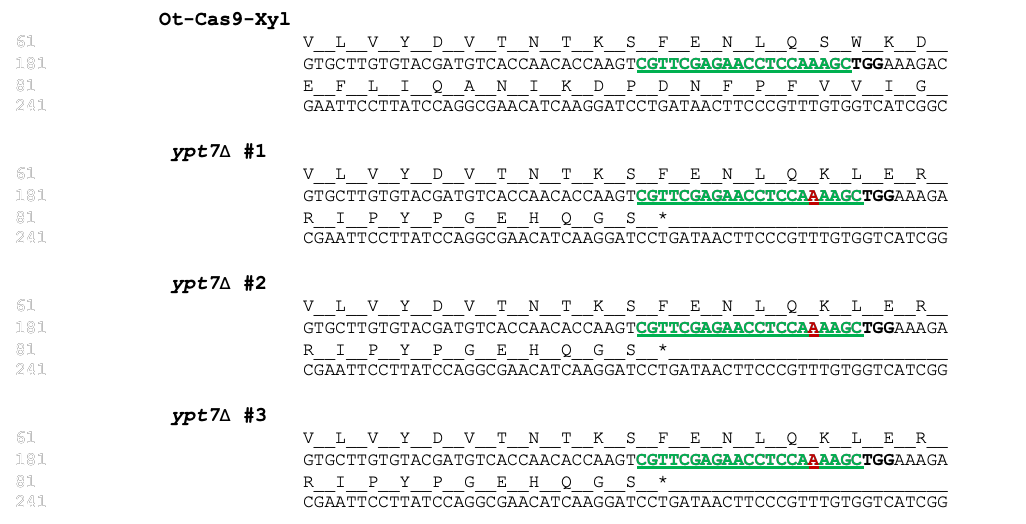


**Figure 4 Nucleotide and translated amino acid sequences of disrupted mutant strains compared to Ot-Cas9-Xyl control.** (A) *ATG12*, (B) *SOD1*, (C) *VPS1* and (D) *YPT7* related genes. The underlined green letter indicates 20-bp specific determinant sequences of gRNA, red letter indicates indel mutations and asterisk (*) indicates stop codon.


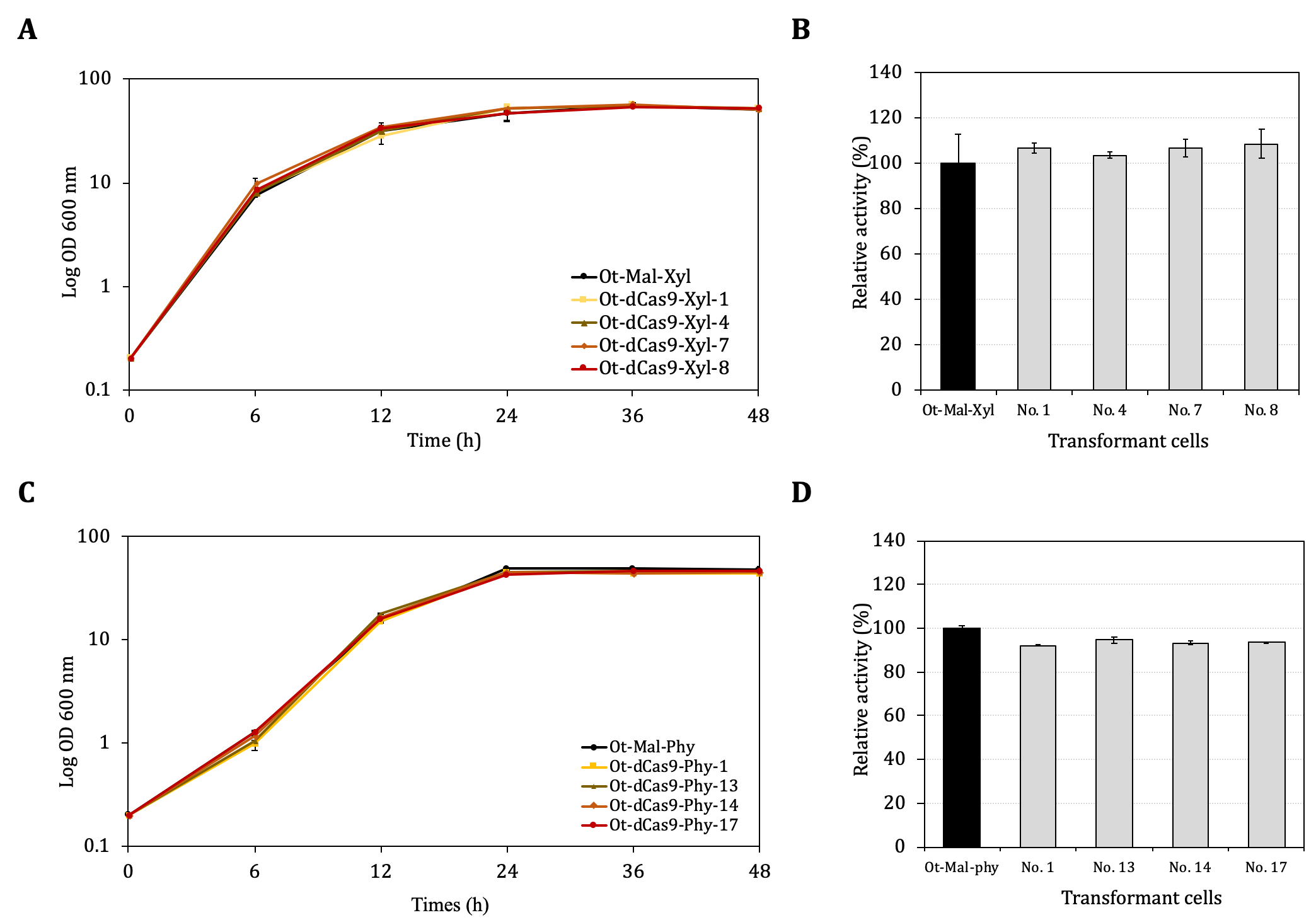


**Figure 5 Growth profiling and enzyme activity.** (A-B) Ot-dCas9-VP64-Xyl and (C-D) Ot-dCas9-VP64-Phy transformants. Data are shown as mean ± S.D. from three-independent biological replicate experiments (*n*=3). Ot-dCas9-VP64-Xyl no. 7 and Ot-dCas9-VP64-Phy no. 14 were then selected to transform with pOtAOX-gRNA in this work.
