# Supplementary material for: Modulation of heterologous protein secretion in the thermotolerant methylotrophic yeast Ogataea thermomethanolica TBRC 656 by CRISPR-Cas9 system
Source: PLoS One. 2021 Sep 28;16(9):e0258005. doi: 10.1371/journal.pone.0258005 (PMC8478189; doi:10.1371/journal.pone.0258005)
Supplement: S2 File — (DOCX) [file pone.0258005.s004.docx]

**Supporting Information**

**Modulation of heterologous protein secretion in the thermotolerant methylotrophic yeast *Ogataea thermomethanolica* TBRC 656 by CRISPR-Cas9 system**

Worarat Kruasuwan^1^, Aekkachai Puseenam^1^, Chitwadee Phithakrotchanakoon^2^,

Sutipa Tanapongpipat^1^ and Niran Roongsawang^1,^*

^1^ Microbial Cell Factory Research Team, Microbial Biotechnology and Biochemicals Research Unit, National Center for Genetic Engineering and Biotechnology, National Science and Technology Development Agency, 113 Thailand Science Park, Phahonyothin Road, Khlong Nueng, Khlong Luang, Pathum Thani 12120, Thailand

^2^ Microbial Systems and Computational Biology Research Team, Thailand Bioresource Research Center, National Center for Genetic Engineering and Biotechnology, National Science and Technology Development Agency, 113 Thailand Science Park, Phahonyothin Road, Khlong Nueng, Khlong Luang, Pathum Thani 12120, Thailand.

***Correspondence author**:

Niran Roongsawang, Ph.D. (NR)

Microbial Cell Factory Research Team, Microbial Biotechnology and Biochemicals Research Unit, National Center for Genetic Engineering and Biotechnology, National Science and Technology Development Agency, 113 Thailand Science Park, Phahonyothin Road, Khlong Nueng, Khlong Luang, Pathum Thani 12120, Thailand.

Tel. +66 2564 6700; Fax. +66 2564 6707

Email: niran.roo@biotec.or.th

**Table 1 List of gRNA cassettes for single gene disruption by CRISPR-Cas9 used in this study.** Sequences of gRNA cassette fragment (HH–20 bp specific determinant sequence–structural gRNA–HDV) with the addition of *Eco*RI and *Kpn*I restriction sites for pOtAOX-gRNA plasmids construction in this study. *Eco*RI (gaattc) and *Kpn*I (ggtacc) sequences are respectively in blue, the 20-bp specific determinant sequences of gRNA is in green, six nucleotides complementary to six nucleotides of targeted promoter sequences is in red and the sequences of HH and HDV ribozymes are in dark blue.

| **Gene** | **gRNA** | **gRNA cassette sequences (5’ to 3’)** |
| --- | --- | --- |
| *ATG12* | Atg12-gRNA1 | gaattcattggtctgatgagtccgtgaggacgaaacgagtaagctcgtccagatccgtttcaaatcgatgttttagagctagaaatagcaagttaaaataaggctagtccgttatcaacttgaaaaagtggcaccgagtcggtgcttttggccggcatggtcccagcctcctcgctggcgccggctgggcaacatgcttcggcatggcgaatgggacggtacc |
|  | Atg12-gRNA2 | gaattcattggtctgatgagtccgtgaggacgaaacgagtaagctcgtcctcttcaattttgcgattccgttttagagctagaaatagcaagttaaaataaggctagtccgttatcaacttgaaaaagtggcaccgagtcggtgcttttggccggcatggtcccagcctcctcgctggcgccggctgggcaacatgcttcggcatggcgaatgggacggtacc |
| *ATG18* | Atg18-gRNA | gaattcattggtctgatgagtccgtgaggacgaaacgagtaagctcgtcgacttctcctgcgtgtctgtgttttagagctagaaatagcaagttaaaataaggctagtccgttatcaacttgaaaaagtggcaccgagtcggtgcttttggccggcatggtcccagcctcctcgctggcgccggctgggcaacatgcttcggcatggcgaatgggacggtacc |
| *SOD1* | Sod1-gRNA | gaattcattggtctgatgagtccgtgaggacgaaacgagtaagctcgtcaccaatggatgtacctccgcgttttagagctagaaatagcaagttaaaataaggctagtccgttatcaacttgaaaaagtggcaccgagtcggtgcttttggccggcatggtcccagcctcctcgctggcgccggctgggcaacatgcttcggcatggcgaatgggacggtacc |
| *VPS1* | Vps1-gRNA | gaattcaacaacctgatgagtccgtgaggacgaaacgagtaagctcgtcgttgttgggtctcaatcctcgttttagagctagaaatagcaagttaaaataaggctagtccgttatcaacttgaaaaagtggcaccgagtcggtgcttttggccggcatggtcccagcctcctcgctggcgccggctgggcaacatgcttcggcatggcgaatgggacggtacc |
| *YPT7* | Ypt7-gRNA | gaattccgaacgctgatgagtccgtgaggacgaaacgagtaagctcgtccgttcgagaacctccaaagcgttttagagctagaaatagcaagttaaaataaggctagtccgttatcaacttgaaaaagtggcaccgagtcggtgcttttggccggcatggtcccagcctcctcgctggcgccggctgggcaacatgcttcggcatggcgaatgggacggtacc |

**Table 2 List of gRNA cassettes for gene activation by CRISPR-dCas9 used in this study.** Sequences of gRNA cassette fragment (HH–20 bp specific determinant sequence–structural gRNA–HDV) with the addition of *Eco*RI and *Kpn*I restriction sites for pOtAOX-gRNA plasmids construction in this study. *Eco*RI (gaattc) and *Kpn*I (ggtacc) sequences are respectively in blue, the 20-bp specific determinant sequences of gRNA is in green, six nucleotides complementary to six nucleotides of targeted promoter sequences is in red and the sequences of HH and HDV ribozymes are in dark blue.

| **Promoter** | **gRNA** | **gRNA cassette sequences (5’ to 3’)** |
| --- | --- | --- |
| *SOD1* | pSod1-gRNA1 | gaattcaaggttctgatgagtccgtgaggacgaaacgagtaagctcgtcaaccttgatgagcaagtttggttttagagctagaaatagcaagttaaaataaggctagtccgttatcaacttgaaaaagtggcaccgagtcggtgcttttggccggcatggtcccagcctcctcgctggcgccggctgggcaacatgcttcggcatggcgaatgggacggtacc |
|  | pSod1-gRNA2 | gaattcaggcatctgatgagtccgtgaggacgaaacgagtaagctcgtcatgccttgcgcatgaggcatgttttagagctagaaatagcaagttaaaataaggctagtccgttatcaacttgaaaaagtggcaccgagtcggtgcttttggccggcatggtcccagcctcctcgctggcgccggctgggcaacatgcttcggcatggcgaatgggacggtacc |
|  | pSod1-gRNA3 | gaattcggggcgctgatgagtccgtgaggacgaaacgagtaagctcgtccgccccgttcgaatgtcaacgttttagagctagaaatagcaagttaaaataaggctagtccgttatcaacttgaaaaagtggcaccgagtcggtgcttttggccggcatggtcccagcctcctcgctggcgccggctgggcaacatgcttcggcatggcgaatgggacggtacc |
|  | pSod1-gRNA4 | gaattcggggcgctgatgagtccgtgaggacgaaacgagtaagctcgtcgattactaattgccaccaatgttttagagctagaaatagcaagttaaaataaggctagtccgttatcaacttgaaaaagtggcaccgagtcggtgcttttggccggcatggtcccagcctcctcgctggcgccggctgggcaacatgcttcggcatggcgaatgggacggtacc |
| *VPS1* | pVps1-gRNA1 | gaattccccaacctgatgagtccgtgaggacgaaacgagtaagctcgtcgttgggaggcgcttggtgtcgttttagagctagaaatagcaagttaaaataaggctagtccgttatcaacttgaaaaagtggcaccgagtcggtgcttttggccggcatggtcccagcctcctcgctggcgccggctgggcaacatgcttcggcatggcgaatgggacggtacc |
|  | pVps1-gRNA2 | gaattcctttgcctgatgagtccgtgaggacgaaacgagtaagctcgtcgcaaaggtagaggtgatttcgttttagagctagaaatagcaagttaaaataaggctagtccgttatcaacttgaaaaagtggcaccgagtcggtgcttttggccggcatggtcccagcctcctcgctggcgccggctgggcaacatgcttcggcatggcgaatgggacggtacc |
|  | pVps1-gRNA3 | gaattcctctcgctgatgagtccgtgaggacgaaacgagtaagctcgtccgagagggtgaccagtagacgttttagagctagaaatagcaagttaaaataaggctagtccgttatcaacttgaaaaagtggcaccgagtcggtgcttttggccggcatggtcccagcctcctcgctggcgccggctgggcaacatgcttcggcatggcgaatgggacggtacc |

**Table 2** (Cont.)

| **Promoter** | **gRNA** | **gRNA cassette sequences (5’ to 3’)** |
| --- | --- | --- |
| *YPT7* | pYpt7-gRNA1 | gaattcgacattctgatgagtccgtgaggacgaaacgagtaagctcgtcaatgtcaatgtggccattccgttttagagctagaaatagcaagttaaaataaggctagtccgttatcaacttgaaaaagtggcaccgagtcggtgcttttggccggcatggtcccagcctcctcgctggcgccggctgggcaacatgcttcggcatggcgaatgggacggtacc |
|  | pYpt7-gRNA2 | gaattccaacttctgatgagtccgtgaggacgaaacgagtaagctcgtcaagttgccggtgagcgttgagttttagagctagaaatagcaagttaaaataaggctagtccgttatcaacttgaaaaagtggcaccgagtcggtgcttttggccggcatggtcccagcctcctcgctggcgccggctgggcaacatgcttcggcatggcgaatgggacggtacc |
|  | pYpt7-gRNA5 | gaattctcagaactgatgagtccgtgaggacgaaacgagtaagctcgtcttctgagtctttggagaagcgttttagagctagaaatagcaagttaaaataaggctagtccgttatcaacttgaaaaagtggcaccgagtcggtgcttttggccggcatggtcccagcctcctcgctggcgccggctgggcaacatgcttcggcatggcgaatgggacggtacc |
|  | pYpt7-gRNA6 | gaattccaaaatctgatgagtccgtgaggacgaaacgagtaagctcgtcattttgggcgactctggagtgttttagagctagaaatagcaagttaaaataaggctagtccgttatcaacttgaaaaagtggcaccgagtcggtgcttttggccggcatggtcccagcctcctcgctggcgccggctgggcaacatgcttcggcatggcgaatgggacggtacc |

**Table 3 Xylanase activity of disrupted mutant yeasts.** Data are shown as mean ± S.D. from three-independent biological replicate experiments (*n*=3).

| **Strain** | **No.** | **Xylanase activity** (U/OD) | **Relative activity** (%U/OD) |
| --- | --- | --- | --- |
| Control | C-1 | 0.61 ± 0.03 | 100.00 |
|  | C-2 | 0.55 ± 0.02 |  |
|  | C-3 | 0.58 ± 0.06 |  |
| *atg12*∆ | 1 | 0.55 ± 0.01 | 94.24 |
|  | 4 | 0.54 ± 0.05 | 93.74 |
|  | 5 | 0.51 ± 0.03 | 87.07 |
| *sod1*∆ | 1 | 0.38 ± 0.09 | 54.05 |
|  | 2 | 0.37 ± 0.06 | 53.19 |
|  | 4 | 0.40 ± 0.07 | 57.10 |
| *vps1*∆ | 1 | 0.46 ± 0.03 | 61.09 |
|  | 2 | 0.45 ± 0.03 | 59.49 |
|  | 3 | 0.45 ± 0.00 | 59.48 |
| *ypt7*∆ | 1 | 0.36 ± 0.01 | 61.46 |
|  | 2 | 0.41 ± 0.01 | 69.61 |
|  | 3 | 0.39 ± 0.02 | 65.30 |

**Table 4 Xylanase activity of Ot-dCas9-VP64-Xyl expressing gRNAs.** Data are shown as mean ± S.D. from three-independent biological replicate experiments (*n*=3).

| **Gene** | **gRNA** | **Xylanase activity** (U/OD) | **Relative activity** (%U/OD) |
| --- | --- | --- | --- |
| Control | C-1 | 1.60 ± 0.12 | 100.00 |
|  | C-2 | 1.74 ± 0.05 |  |
|  | C-3 | 1.65 ± 0.04 |  |
| *SOD1* | 1 | 2.42 ± 0.18 | 122.84 |
|  | 2 | 2.33 ± 0.19 | 118.27 |
|  | 3 | 2.27 ± 0.13 | 115.23 |
|  | 4 | 2.05 ± 0.11 | 104.06 |
| *VPS1* | 1 | 1.94 ± 0.14 | 111.32 |
|  | 2 | 2.26 ± 0.21 | 129.87 |
|  | 3 | 2.08 ± 0.18 | 119.68 |
| *YPT7* | 1 | 1.98 ± 0.10 | 120.04 |
|  | 2 | 1.89 ± 0.09 | 114.66 |
|  | 5 | 2.05 ± 0.48 | 124.15 |
|  | 6 | 1.69 ± 0.07 | 102.43 |

**Table 5 Relative gene expression level of Ot-dCas9-VP64-Xyl expressing gRNAs.** *ACT* was used to normalize gene expression. Data are shown as mean ± S.D. from three-independent biological replicate experiments (*n*=3).

| **Gene** | **gRNA** | **Fold change** (FC) | **Relative expression** (%FC) |
| --- | --- | --- | --- |
| Control |  | 1.00 ± 0.05 | 100.00 |
| *SOD1* | 1 | 1.48 ± 0.23 | 148.15 |
|  | 2 | 1.20 ± 0.26 | 119.81 |
|  | 3 | 1.18 ± 0.21 | 118.12 |
|  | 4 | 1.05 ± 0.04 | 104.64 |
| *VPS1* | 1 | 1.27 ± 0.19 | 127.30 |
|  | 2 | 1.33 ± 0.18 | 133.48 |
|  | 3 | 1.24 ± 0.20 | 124.89 |
| *YPT7* | 1 | 1.15 ± 0.08 | 115.28 |
|  | 2 | 1.22 ± 0.11 | 122.12 |
|  | 5 | 1.37 ± 0.28 | 137.01 |
|  | 6 | 1.06 ± 0.14 | 106.08 |

**Table 6 Phytase activity of Ot-dCas9-VP64-Phy expressing gRNAs.** Data are shown as mean ± S.D. from three-independent biological replicate experiments (*n*=3).

| **Gene** | **gRNA** | **Phytase activity** (U/OD) | **Relative activity** (%U/OD) |
| --- | --- | --- | --- |
| Control | C-1 | 0.31 ± 0.02 | 100.00 |
|  | C-2 | 0.31 ± 0.00 |  |
|  | C-3 | 0.43 ± 0.05 |  |
| *SOD1* | 1 | 0.36 ± 0.02 | 106.69 |
|  | 2 | 0.37 ± 0.01 | 104.19 |
|  | 3 | 0.39 ± 0.01 | 104.59 |
| *VPS1* | 1 | 0.44 ± 0.00 | 125.39 |
|  | 2 | 0.42 ± 0.02 | 120.63 |
|  | 3 | 0.38 ± 0.02 | 109.13 |
| *YPT7* | 1 | 0.39 ± 0.02 | 110.56 |
|  | 2 | 0.41 ± 0.02 | 116.14 |
|  | 5 | 0.40 ± 0.02 | 114.79 |

**Table 7 Relative gene expression level of Ot-dCas9-VP64-Phy expressing gRNAs.** *ACT* was used to normalize gene expression. Data are shown as mean ± S.D. from three-independent biological replicate experiments (*n*=3).

| **Gene** | **gRNA** | **Fold change** (FC) | **Relative expression** (%FC) |
| --- | --- | --- | --- |
| Control |  | 1.00 ± 0.12 | 100.00 |
| *SOD1* | 1 | 1.25 ± 0.20 | 125.27 |
|  | 2 | 1.13 ± 0.16 | 112.66 |
|  | 3 | 1.05 ± 0.12 | 105.06 |
| *VPS1* | 1 | 1.31 ± 0.08 | 130.94 |
|  | 2 | 1.09 ± 0.20 | 109.26 |
|  | 3 | 1.15 ± 0.10 | 114.70 |
| *YPT7* | 1 | 1.17 ± 0.19 | 117.26 |
|  | 2 | 1.05 ± 0.11 | 105.25 |
|  | 5 | 1.03 ± 0.15 | 102.78 |
