# Supplementary material for: Modulation of heterologous protein secretion in the thermotolerant methylotrophic yeast Ogataea thermomethanolica TBRC 656 by CRISPR-Cas9 system
Source: PLoS One. 2021 Sep 28;16(9):e0258005. doi: 10.1371/journal.pone.0258005 (PMC8478189; doi:10.1371/journal.pone.0258005)
Supplement: S2 Raw images — (PDF) [file pone.0258005.s006.pdf]

A

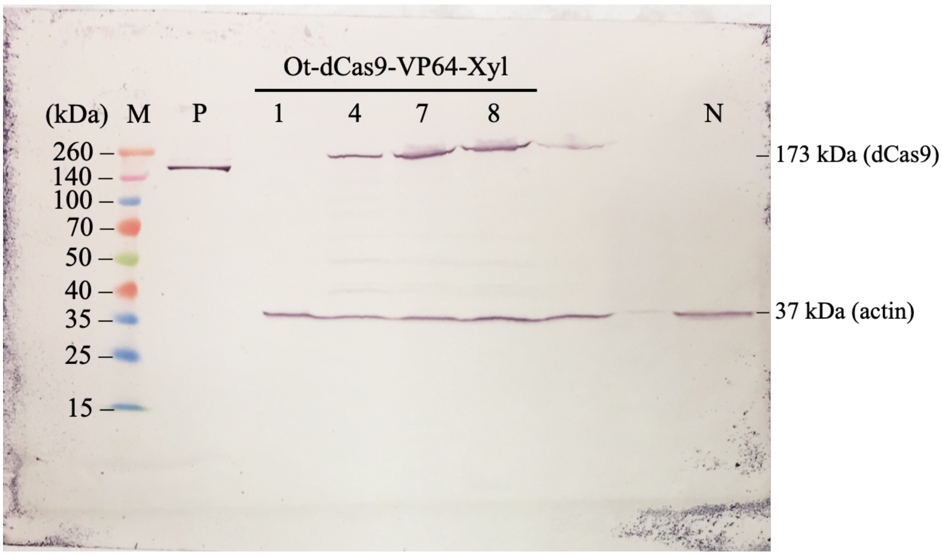

B

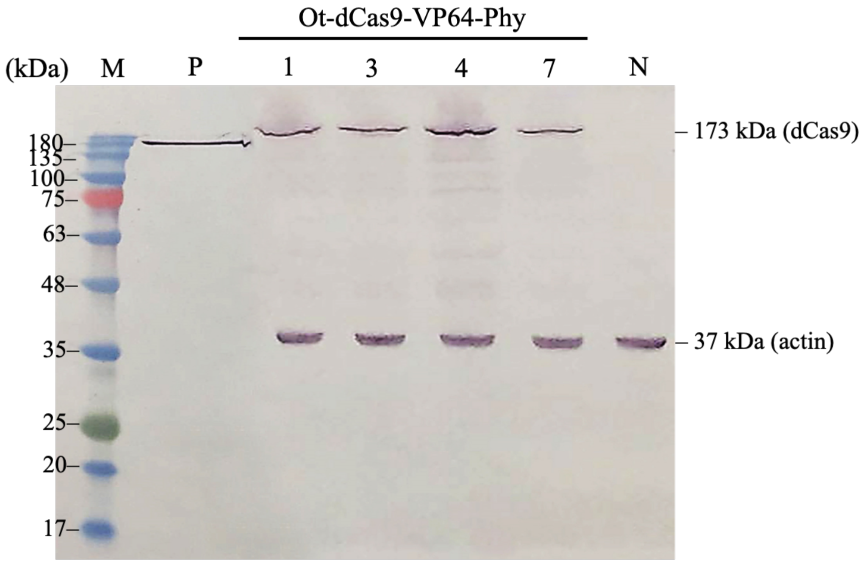

S2 Raw images. Western blot analysis. dCas9-VP64 expressed band (173 kDa) of (A) Ot-dCas9-VP64-Xyl and (B) Ot-dCas9-VP64-Phy transformants. Either *O. thermomethanolica* expressing xylanase (Ot-Mal-Xyl) or phytase (Ot-Mal-Phy) used as a negative control (N) under YPS condition. The protein probed by monoclonal anti-Cas9 and anti-actin. Lane M, pre-stained protein molecular marker; purified Cas9 was loaded as positive control (P).
